# Oncogenic potential of truncated-Gli3 via the Gsk3β/Gli3/AR-V7 axis in castration-resistant prostate cancer

**DOI:** 10.1038/s41388-024-03266-z

**Published:** 2025-01-16

**Authors:** Jyoti B. Kaushal, Pratima Raut, Sushanta Halder, Zahraa W. Alsafwani, Seema Parte, Gunjan Sharma, K. M. Abdullah, Parthasarathy Seshacharyulu, Subodh M. Lele, Surinder K. Batra, Jawed A. Siddiqui

**Affiliations:** 1 Department of Biochemistry and Molecular Biology, University of Nebraska Medical Center, Omaha, NE USA; 2 Department of Cell and Molecular Biology, University of Mississippi Medical Center, Jackson, MS USA; 3 Department of Pathology and Microbiology, University of Nebraska Medical Center, Omaha, NE USA; 4 Fred and Pamela Buffett Cancer Center, University of Nebraska Medical Center, Omaha, NE USA; 5 Eppley Institute for Cancer and Allied Diseases Research, University of Nebraska Medical Center, Omaha, NE USA; 6 Cancer Center Research Institute, University of Mississippi Medical Center, Jackson, MS USA

**Keywords:** Prostate cancer, Apoptosis

## Abstract

The functional activation of the androgen receptor (AR) and its interplay with the aberrant Hh/Gli cascade are pivotal in the progression of castration-resistant prostate cancer (CRPC) and resistance to AR-targeted therapies. Our study unveiled a novel role of the truncated form of Gli (t-Gli3) in advancing CRPC. Investigation into Gli3 regulation revealed a Smo-independent mechanism for its activation. Despite lacking a transactivation domain, t-Gli3 relies on androgen receptor variant 7 (AR-V7) for its action. Mechanistically, Gsk3β activation led to the t-Gli3 generation, and inhibition of Gsk3β supported the accumulation of full-length Gli3 expression through a non-canonical mechanism. Knockdown of Gsk3β (Gsk3β KD) reduces CRPC cell proliferation, induces apoptosis via mitochondrial fragmentation, and triggers metabolomic reprogramming. The in vivo studies with Gsk3β KD cells in the mouse prostate resulted in tumor growth retardation compared to scramble cells. RNA-seq HALLMARK Gene Set Enrichment Analysis (GSEA) analysis of Gsk3β KD revealed a positive enrichment of apoptosis, tumor suppressor gene, and negative enrichment of oncogenic pathway. Furthermore, combinational use of a Gsk3β inhibitor with anti-Smo or Gli1 significantly inhibited the CRPC cell growth, which is resistant to individual Smo or Gli1 inhibitor targeting. Intriguingly, solely targeting Gli3 showed effectiveness in inhibiting CRPC cell growth. Overall, our study underscores the clinical significance of Gli3, emphasizing t-Gli3, and provides novel insights into the interplay of the Gsk3β/t-Gli3/AR-V7 axis in CRPC.

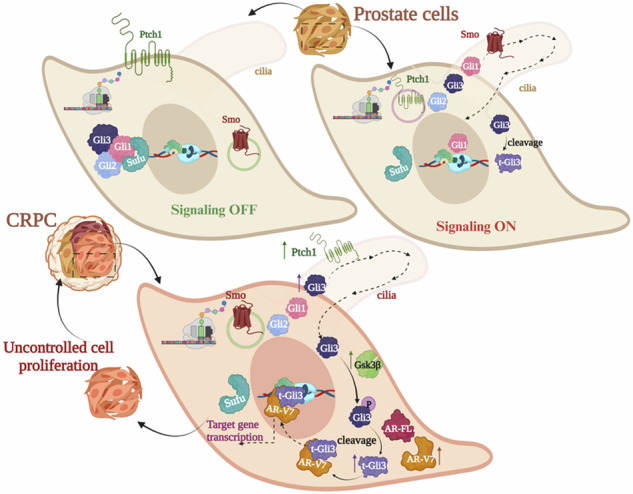

## Introduction

Prostate cancer (PCa) is the most prevalent non-cutaneous cancer among men. PCa experienced a 3% annual rise in incidence from 2014 to 2019, breaking a two-decade-long declining trend and resulting in 99,000 new cases [1]. The clinical and molecular phenotypes of PCa display substantial heterogeneity and are influenced by androgenic steroids where the androgen receptor (AR) plays a crucial role [2, 3]. Hormonal therapies targeting the AR pathway are the mainstay of local, advanced, and metastatic forms of PCa [4]. Unfortunately, patients develop castration-resistant prostate cancer (CRPC), where the cancer becomes resistant to hormonal therapies and typically relapses within a year [5]. This resistance can arise from various factors, such as AR mutations, amplification, intratumoral androgen synthesis, and alternative signaling pathways [5, 6]. However, the precise molecular underpinning for the intrinsic or acquired resistance to these AR-targeted therapies is yet to be fully understood and defined.

Emerging evidence on PCa has uncovered the aberrant activation of the Hedgehog (Hh) and Glioma-Associated Oncogene (Gli) signaling cascade, emphasizing its potential role in disease advancement and therapy resistance [7–9]. These effects are mediated through autocrine and paracrine Hh signaling within the tumor microenvironment (TME). Paracrine Hh signaling also stimulates steroidogenesis in benign stromal cells within the TME, promoting prostate tumor growth [10]. While the preclinical studies have demonstrated the potential of Hh inhibitors, especially Smo-inhibitors, to decrease the invasiveness and metastatic potential of PCa, there is still a lack of clinical proof [11, 12].

Gli is a vital transcription factor of the Hh signaling pathway. The Gli-family comprises three proteins (Gli1, Gli2, and Gli3), all with conserved C2–H2 zinc finger domains that modulate the target genes expression, impacting both the developmental process and oncogenesis [13, 14]. In brief, Gli1 serves as a transcriptional activator, while Gli2 acts as a dual-function transcriptional activator/repressor in the presence of Hh. On the other hand, Gli3 predominantly functions as a transcriptional repressor in the absence of Hh signaling. The modulation of these factors primarily occurs through both canonical and non-canonical signaling pathways, rendering the Hh/Smo/Gli cascade highly significant and responsive in cancer [15, 16]. In non-canonical Hh signaling, the components outside the conventional Hh-Ptch-Smo-Gli paradigm play a vital role in Gli transcription. In this context, Gsk3β emerges as a critical player in facilitating this post-translational modification process of Gli proteins [17, 18].

Considering the role of Gli proteins in PCa, high Gli1 expression has been associated with advanced primary tumor stage, positive lymph node metastasis, and advanced clinical stage [19]. Additionally, Gli1 expression positively correlates with stemness markers, particularly CD44, suggesting its potential as a cancer stem cell marker and diagnostic marker for PCa [19]. Hh signaling supports androgen signaling and androgen-independent PCa growth by interacting with two key proteins, Gli2 and AR [20, 21]. Gli2 is overexpressed in CRPC, and its C-terminal domain (CTD) is sufficient for AR co-activation, driving CRPC development [22]. The non-canonical activation of Hh in PCa cells is mediated through the interaction between the transcriptionally active AR and Gli3. This interaction hinders the binding of βTrCP, a ubiquitin ligase, with Gli3, consequently inhibiting the proteolytic processing of Gli3 into its repressor form [23]. Gli3 interacts and functionally cooperates with AR to enrich an AR-dependent gene expression and contributes to castration-resistant tumorogenesis [24]. Mechanistically, the Hh proteins released by androgen-deprived PCa cells stimulate intratumoral steroidogenesis in benign stromal cells within the TME and promote tumor growth [10].

Current investigations collectively highlight the mysterious and unique attributes of Gli-family proteins, which raises the question of which Gli-member is most influential. Furthermore, exploring how Gsk3β activation sustains Gli transactivation through a non-canonical regulatory mechanism, especially in CRPC, where Gsk3β is connected to hormone-independent AR-mediated gene expression and is mainly expressed in high-risk PCa. Thus, the present study aims to unravel the significance of Gli-family proteins, their association with Gsk3β, their biological relevance, and their therapeutic potential in CRPC.

## Results

### High Gli3 expression, especially t-Gli3, is associated with advanced prostate cancer

**Fig. 1:**
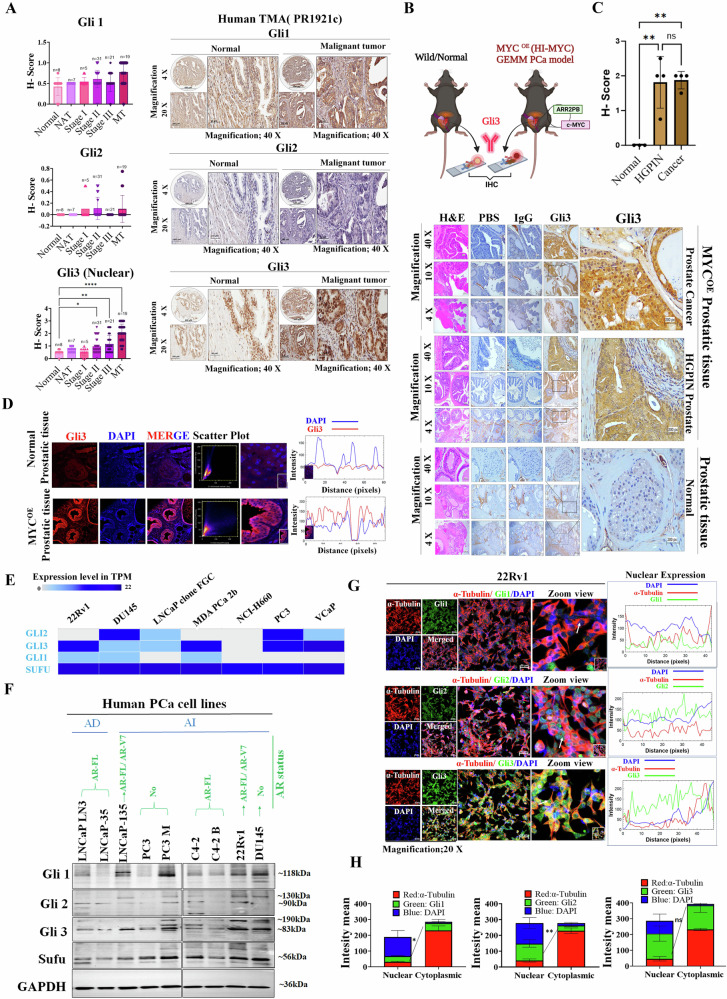
Expression profile Gli-family proteins, with emphasis on t-Gli3 in advanced PCa. **A** Quantitation-H score (left panel) and Representative images (right panel) from IHC showing Gli-family proteins, including Gli1, Gli2, and Gli3 in human TMA of PCa. **B** Schematic representation of MYC^OE^ (Hi-MYC) mouse PCa model **C** IHC quantitation-H score (upper panel) and corresponding representative images demonstrate the expression of Gli3 in mouse MYC^OE^ cancerous prostatic tissue, HGPIN lesions, and age-matched normal mouse prostatic tissue. **D** Representative IF images (left panel) and fluorescence intensity plot (right panel) of Gli3 in mouse MYC^OE^ cancerous prostatic tissue and age-matched normal mouse prostatic tissue. **E** Gene expression of Gli-family proteins and regulator Sufu in human PCa cell lines using expression atlas (https://www.ebi.ac.uk/gxa/home). **F** Representative WB images show the expression of Gli proteins and Sufu invarious PCa cell lines. GAPDH was used as a loading control. **G** Representative IF images (left panel) and fluorescence intensity plot (right panel) of Gli1, Gli2, and Gli3 with α-tubulin in human CRPC cells (22Rv1). **H** The quantitation of intensity mean of nuclear vs. cytoplasmic expression was analyzed using ImageJ software.

To investigate a potential key player among Gli-family proteins, which could serve as a promising candidate for targeting CRPC, we determined the expression of Gli-family proteins using human tissue microarray (TMA) comprising normal, normal adjacent tissue, PRAD (grade I, II, III, and IV), and malignant (unspecified stage). Interestingly, clinical PCa samples revealed significantly increased expression of Gli3 in all malignant stages compared to normal prostate tissue (Fig. 1A). While Gli1 exhibited high expression exclusively in stage II, Gli2 expression remained unchanged compared to normal prostate tissues. The characterization of Gli-family protein expression patterns in malignant clinical samples showed distinct localization of these proteins within the cytosolic and nuclear compartments. Gli1 predominantly resided in the cytosol, Gli2 showed nuclear accumulation, and Gli3 was observed in both cytosolic and nuclear compartments (Fig. 1A). The significantly elevated expression of Gli3 was noted in the nuclear region compared to the normal tissue. Conversely, the changes in Gli3 expression in the cytosolic region were non-significant compared to normal prostate tissues (Fig. S1A). The nuclear accumulation of Gli3 suggests a potential activation of Gli3-mediated signal transduction, contributing to the progression of PCa, especially in advanced stages. We further validated the functional significance of Gli3 localization (nuclear versus cytoplasmic) in human PCa using isotype controls alongside positive and negative tissue controls. We conducted an in silico analysis of Gli3 protein expression through the Human Protein Atlas (Fig. S1B, https://www.proteinatlas.org). Our IHC results revealed high nuclear Gli3 expression in malignant colon tissue, moderate nuclear Gli3 expression in malignant lung tissue, and very low nuclear Gli3 expression in malignant liver tissue (Figure S1C), reinforcing the findings from the Gli3 protein atlas. Consistently, PCa samples from the MYC^OE^ PCa mouse model, which recapitulates the molecular features of human prostate tumors [25], also exhibited increased expression of Gli3, particularly in the nuclear compartment of both high-grade lesion and carcinoma prostatic tissues compared to age-matched normal prostatic tissues (Fig. 1B–D). Our IHC analysis showed mild Gli3 expression in the mouse prostate, higher levels in the spleen and kidney, and no expression in the pancreas and liver (Fig. S2A–B).

Next, considering Sufu serves as the crucial interacting partner of Gli proteins, influencing their actions (GeneCards/STRING interaction network: Fig. S3A), we examined the expression of Gli proteins and Sufu in datasets from human PCa cell lines using an expression atlas. Intriguingly, Gli3 and Sufu exhibited higher expression levels than Gli1 and Gli2 in most PCa cell lines (Fig. 1E). To delve deeper, we looked at the protein expression of Gli1, Gli2, Gli3, and Sufu in various PCa cell lines (Fig. 1F). These PCa cells exhibit varying androgen responses, androgen receptor (AR) expression patterns, and distinct morphology (Fig. S3B). Gli1 and Gli2 expression levels were decreased, while Gli3 expression level was increased, and Sufu expression levels showed no difference in most of the PCa cell lines (Fig. 1F). Surprisingly, the expression of truncated-Gli3 (t-Gli3) was found to be increased in all PCa cell lines, with some showing abundant expression of both full-length (FL-Gli3~190 kDa) and t-Gli3 (~83 kDa) Gli3 proteins, such as PC3M and 22Rv1. Moving forward, experiments with mouse syngeneic cell lines demonstrated increased expression of both (full-length and truncated) forms of Gli3 in castrated PTEN conditional knockout mouse-derived cells (cE2) compared to non-castrated mouse-derived cells (E2) (Fig. S3C). These observations suggest the potential involvement of t-Gli3 in an advanced stage of PCa progression, including metastasis and CRPC.

The specific localization of Gli proteins in the sub-cellular compartment was confirmed using 22Rv1 human PCa cell line (resistant to enzalutamide and regarded as CRPC cell line [26, 27]) via immunofluorescence imaging with Gli1, Gli2, and Gli3 along with α-tubulin, a cytoskeletal protein (Fig. 1G). Nuclear localization of Gli proteins was represented via fluorescence intensity profile (Fig. 1G right panel). We also quantified the cytosolic vs. nuclear expression of Gli proteins (Fig. 1H). These cumulative results suggested that the cytosolic expression of Gli1, nuclear expression of Gli2, and Gli3 are consistent with the localization pattern observed in human TMA of PCa patients. Considering the expression pattern of Gli-family proteins, observation revealed that Gli3 might have a more potent role in the advanced PC context; however, further evidence is needed to validate this hypothesis.

### Gli3 is modulated with androgen deprivation and treatment with AR agonist/antagonist

**Fig. 2:**
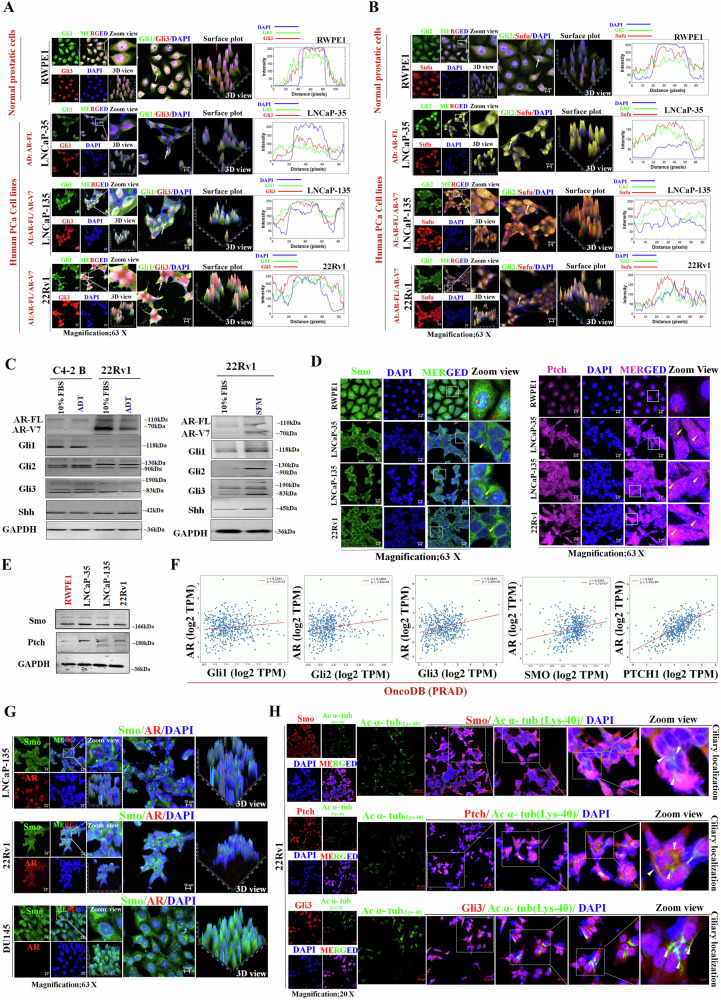
Sub-cellular expression profile of Gli-family proteins with the dependency of AR and ciliary localization of Hh receptors. **A**, **B** Representative IF images for sub-cellular localization of Gli1/Gli3 and Gli2/ Sufu (left panel) and fluorescence intensity plot (right panel) in normal human prostate cells (RWPE1) and PCa cell lines (AD: LNCaP-35; AI: LNCaP-135 & 22Rv1). **C** Representative WB images depict the expression of Shh/Gli cascade proteins under ADT (C4-2B and 22Rv1) and SFM conditions (22Rv1). GAPDH was used as a loading control. **D** Representative IF images of Smo (left panel) and Ptch (right panel) in normal human prostate cells and PCa cell lines with the status of AD and AI. Arrowheads mark the localized proteins, red for nuclear and yellow for cytoplasmic area. **E** Representative WB images show the expression of Smo and Ptch receptors in normal human prostate cells RWPE1 and PCa cell lines (AD: LNCaP-35; AI: LNCaP-135 & 22Rv1). **F** A scatter plot depicting the correlation of Gli-family transcription factor (Gli1, Gli2, and Gli3), and Hh receptors (Smo and Ptch1) with AR in TCGA-PRAD patients using OncoDB. **G** Representative IF images for colocalization analysis of Smo and AR in PCa cell lines display AR presence (LNCaP-135 and 22Rv1) and absence (DU145), along with AI status. **H** Representative IF images for ciliary localization of Smo, Ptch, and Gli3, where cilia were examined using acetylated α-tubulin. Arrowheads mark the localization of proteins, whether colocalized or not.

In the subsequent analysis, the investigation of sub-cellular localization of Hh signaling regulatory proteins, including Gli1, Gli2, Gli3, and Sufu, revealed increased expression of Gli3 (both cytosolic and nuclear) and Sufu (cytosolic) in androgen-independent (AI)/CRPC cell lines compared to androgen-dependent (AD) and normal prostate cell lines (Fig. 2A, B). Notably, Gli1 expression was reduced in AD cell lines but unchanged in AI/CRPC cell lines. In contrast, the Gli2 expression was increased in AD cell lines but decreased in AI/CRPC cell lines. To demonstrate the effect of androgen-deprived conditions on the Shh/Gli cascade, we utilized PCa cells expressing either AR-FL (C4-2B) or AR-FL/AR-V7 (22Rv1). Under the androgen-deprived condition, the expression of Gli1, Gli2, and Shh proteins showed no alterations, but AR and Gli3, exclusively t-Gli3 expression, were downregulated in 22Rv1 cells, but not in C4-2B cells (Fig. 2C). This indicates the involvement of the non-canonical Hh pathway, specifically ligand-independent Hh signaling, in response to ADT due to the presence of AR-V7 in 22Rv1. Further, the cytosolic and nuclear fractions revealed the diminished nuclear accumulation of AR-FL/AR-V7 (Fig. S4A). In contrast, under serum-free media (SFM) conditions, the upregulated expression of AR, Shh, Gli1, Gli2, and Gli3 was observed in 22Rv1 cells (Fig. 2C). These results support the involvement of the canonical Hh pathway, precisely the ligand-dependent Shh/Gli axis, in response to SFM-induced starvation. Considering the differential expression pattern of Gli3 in different AR-positive and negative PCa cell lines, we next investigated Gli3 activity in relation to AR signaling. We treated the normal prostatic epithelial cell line (RWPE1), the AR-FL-only expressing cell line (C4-2B), and the cell line co-expressing AR-FL and AR-V7 (22Rv1) with AR antagonists (ENZ-Enzalutamide), agonists (DHT - Dihydrotestosterone) or a combination of both and determined AR and Gli3 expression. We observed the upregulation of AR and Gli3 in RWPE1 and C4-2B cell lines treated with DHT, while treatment with ENZ or a combination led to their downregulation (Fig. S4B). No effect was seen in the 22Rv1 cell line, which is considered an ENZ-resistant cell line (Fig. S4B). Confocal microscopy provided additional evidence that aligned with the expression patterns seen in WB, reinforcing the notion that the presence of AR is crucial for sustaining Gli3 expression (Fig. S4C). Additionally, we observed increased Gli3 expression, along with downregulation of Gli1 and Gli2, and no changes in Shh in C4-2B ENZ-resistant cells compared to parental C4-2B cells, suggesting Gli3’s role in advanced disease progression (Fig. S4D).

### Ciliary localization of Ptch1 is associated with Gli3 activation via a Smo-independent mechanism

Unraveling the canonical regulation of Gli3 protein, influenced by receptor-mediated activation of the Hh pathway, the expression level of Smo and Ptch in both AD and AI PCa cells along with normal prostate cells were examined. The predominantly membranous expression of Smo in PCa cells was observed in contrast to normal cells exhibiting Smo expression in both the cytosolic and nuclear regions (Fig. 2D). Intriguingly, Ptch displayed an inverse expression pattern localized in both cytosolic and nuclear regions in AD and AI PCa cells compared to normal prostate cells. As Ptch acts as a downstream target of Gli, the elevated expression of Ptch in PCa cells indicates that this overexpression could result from Gli3 activation. We observed a reduction in Smo expression and an elevation in Ptch expression in PCa cell lines compared to normal prostate cells (Fig. 2E). Furthermore, we performed a correlation analysis using OncoDB to examine the relationship between AR and Gli-family proteins (Gli1, Gli2, and Gli3) and receptors (Smo and Ptch1). Interestingly, we observed a positive correlation specifically between Gli3/AR (weak) and Ptch1/-AR (moderate) at the gene expression level of PRAD patients (Fig. 2F). In the same cohorts, no significant correlation was found between AR and Gli1 or Gli2 or Smo (Fig. 2F). This further emphasizes the potential relevance of Gli3 with functional activation of AR in the PCa. In support, our investigation into the association of Smo with AR also revealed no colocalization of Smo and AR in CRPC cells, suggesting a lack of direct association between these proteins (Fig. 2G). Notably, we found reduced Gli3 nuclear expression in DU145, which does not express AR, suggesting the nuclear translocation of Gli3 may be due to the presence of AR (Fig. 2G).

Further, we investigated the presence of Smo, Ptch, and Gli3 on cilia, which is a crucial determinant for receptor-mediated modulation of the Smo/Gli canonical axis. A subset of PCa cells exhibited cilia and the Ptch expression on the cilium. Strikingly, no expression of Smo and Gli3 on the cilium was observed (Fig. 2H). The absence of Smo on the cilium, coupled with the presence of Ptch, provides additional evidence supporting the involvement of a Smo-independent non-canonical mechanism. Additionally, the lack of Gli3 expression on the cilium and the presence in the nucleus suggest the role of Gli3 as a transcription factor activating Hh target proteins in CRPC cells (Fig. 2H).

### Androgen receptor variant (AR-V7) is required for Gli3-mediated action

**Fig. 3:**
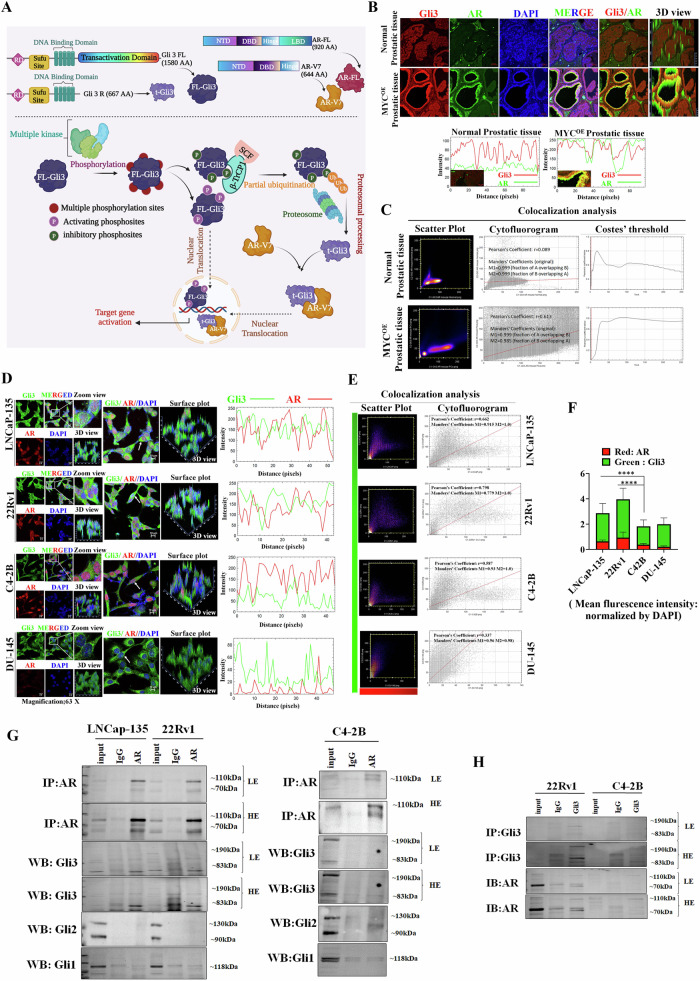
Colocalization of AR and Gli3 and an association between AR-V7 and t-Gli3. **A** Schematic representation of biochemical domains of FL-Gli3, t-Gli3, AR, and AR-V7 and hypothesized possible signaling mechanism. **B** Representative IF images (upper panel) and fluorescence intensity plot (lower panel) of Gli3 and AR in mouse MYC^OE^ cancerous and normal prostatic tissues. **C** Colocalization analysis using ImageJ software using plugins (colocalization finder and JACoP). **D** Representative IF images (left panel) and fluorescence intensity plot (right panel) in normal and PCa cell lines, including LNCaP-135 and 22Rv1 expressing AR-FL/AR-V7, C4-2B expressing AR, and DU145 expressing no AR. **E** Colocalization analysis using ImageJ software using plugins (colocalization finder and JACoP). **F** The intensity mean of AR and Gli3 was quantified using ImageJ software. **G** Representative WB image of Gli1, Gli2, and Gli3 after AR enrichment via IP in LNCaP-135, 22Rv1, and C4-2B cells **H** Representative WB image of Gli3 and AR after Gli3 enrichment via IP in 22Rv1 and C4-2B cells.

To understand the role of Gli3 for downstream target activation and its impact on CRPC, we analyzed the biochemical structures of Gli3 (FL-Gli3 and t-Gli3) and AR (AR-FL and AR-V7), which led to an intriguing observation (Fig. 3A). FL-Gli3 possesses a transactivation domain, whereas t-Gli3 lacks this domain. The elevated AR expression was colocalized with increased Gli3 expression (Fig. 3B) with a Pearson coefficient of 0.6 (Fig. 3C), indicating a high correlation between AR and Gli3 in MYC^OE^ prostatic tissue (Fig. 3C). A prominent colocalization of AR and Gli3 was observed in the LNCaP-135 and 22Rv1 cell lines expressing both AR-FL and AR-V7 variants (Fig. 3D). However, there was no Gli3 localization in the nucleus of C4-2B cells, expressing only AR-FL and the DU145 cells with the lack of AR expression. In addition, our colocalization analysis inspected correlation coefficients in 22Rv1 (*r* = 0.6) and LNCaP-135 (*r* = 0.7) showed a strong correlation between both proteins (Fig. 3E). Moreover, analysis of quantitative mean fluorescence intensity suggested Gli3 expression is positively regulated by AR expression, where more pronounced AR and Gli3 expression in 22Rv1 and LNCaP-135 as compared to C4-2B, suggesting that AR-V7 might be a regulator of FL-Gli3 and t-Gli3 (Fig. 3F).

To investigate the potential interaction between t-Gli3 and AR-V7, we performed a co-immunoprecipitation (co-IP) assay with AR enrichment using cell lines expressing both AR-FL and AR-V7 (LNCaP-135 and 22Rv1) and the AR-FL-only expressing cell line C4-2B (Fig. 3G). Our findings revealed an interaction between t-Gli3 and AR-V7 in LNCaP-135 and 22Rv1 cells (Fig. 3G left panel). Interestingly, no interaction was observed in C4-2B cells lacking AR-V7 (Fig. 3G right panel). This result suggests a selective interaction between t-Gli3 and AR-V7 in CRPC cells, further validated by performing a reverse co-IP approach (Fig. 3H). This additional evidence strengthens the observation of a specified interaction between t-Gli3 and AR-V7, reinforcing the significance of this interaction in the context of CRPC.

### Gsk3β- mediated non-canonical mechanism involved in Gli3 processing

**Fig. 4:**
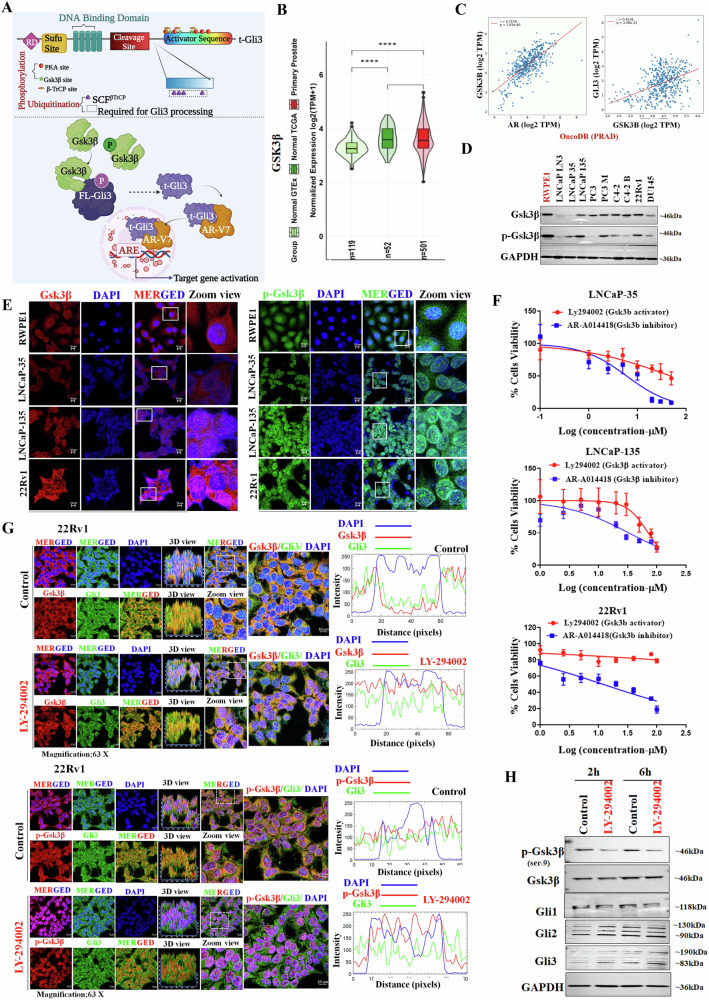
Gsk3β- mediated non-canonical mechanism for Gli3 processing. **A** Schematic representation of biochemical domains of Gli3 consisting of the post-translational binding site and proposed mechanism. **B** Gsk3β expression level in primary tumor vs. normal prostate human samples (GTEx and TCGA). **C** A scatter plot of correlation analysis of Gsk3β/AR and Gsk3β/Gli3genes using in silico approach in TCGA-PRAD patients using OncoDB. **D** Representative WB images show the expression of Gsk3β and p-Gsk3β in normal human prostate cells and various PCa cell lines. GAPDH was used as a loading control. **E** Representative IF images showing the expression of Gsk3β (left panel) and p-Gsk3β (right panel) in normal human prostate cells RWPE1 and PCa cell lines (AD: LNCaP-35; AI: LNCaP-135 & 22Rv1). **F** Cell viability assay using Gsk3β-activator and inhibitors in AD and AI PCa cell lines. Values are expressed as mean ± SEM (*n* = 5). **G** Representative IF images (left panel) and fluorescence intensity plot (right panel) showing sub-cellular expression Gsk3β/Gli3 (upper panel) and p-Gsk3β/Gli3 (lower panel) in 22Rv1 cells after treating Gsk3β-activator. **H** Representative WB images show the expression of p-Gsk3β, Gsk3β, and Gli proteins in 22Rv1 cells treated with Gsk3β-activator. GAPDH was used as a loading control.

The biochemical structure of Gli3-FL reveals the presence of a Gsk3β binding site within the activator sequence (Fig. 4A). The expression of Gsk3β is significantly upregulated in PCa patients (GTEx and TCGA) (Fig. 4B). Moreover, a significant positive correlation was observed between Gsk3β/AR (strong) and Gsk3β/Gli3 (moderate) in TCGA-PRAD patients (Fig. 4C). We observed distinct expression patterns of Gsk3β and p-Gsk3β across different PCa cell lines based on their respective AR statuses (Fig. 4D). Furthermore, we investigated the sub-cellular expression of Gsk3β and p-Gsk3β in normal, AD, and AI PCa cell lines. Gsk3β was found to be localized in both cytosolic and nuclear regions of PCa cells (Fig. 4E left panel), whereas p-Gsk3β exhibited predominant nuclear localization (Fig. 4E right panel). In contrast, RWPE1 displayed both cytosolic and nuclear expression of Gsk3β and p-Gsk3β. Importantly, LNCaP-135 and 22Rv1 showed higher expression levels of Gsk3β and p-Gsk3β than LNCaP-35 and RWPE1 cells, suggesting their potential relevance in CRPC. Similarly, Gsk3β expression in human TMA significantly increased in PRAD patients (Fig. S5A).

To explore the functional relevance of Gsk3β in CRPC, we employed a selective Gsk3β activator (LY-294002) and inhibitor (AR-A014418) and investigated the impact of Gsk3β on PCa cells viability (Fig. 4F). Interestingly, we observed a substantial decrease in cell survival when Gsk3β was inhibited. Surprisingly, activating Gsk3β in the AI cell lines (LNCaP-135 and 22Rv1) did not impact cell viability, whereas AD cells (LNCaP-35) showed effect after treatment. To explore the Gsk3β/Gli3 axis, using Gsk3β activator, we observed the significant effect on Gsk3β activation, leading to the dephosphorylation of p-Gsk3β at 2 h and 6 h time points with 10 µM concentration (Fig. S5B). Using confocal microscopy, we noticed the increased expression of both Gsk3β and Gli3, with a predominant nuclear translocation, in Gsk3β activator- treated cells (Fig. 4G upper panel). At the same time, p-Gsk3β expression was decreased and localized only in the nucleus (Fig. 4G lower panel). Next, we observed an increase in Gli3 expression, particularly t-Gli3, after 2 h, and both the full and truncated forms of Gli3 after 6 h (Fig. 4H). These findings suggest that the activity of Gsk3β contributes to the generation of the t-Gli3 protein.

### Gsk3β-knockdown induces an anti-proliferative response in CRPC Cells

**Fig. 5:**
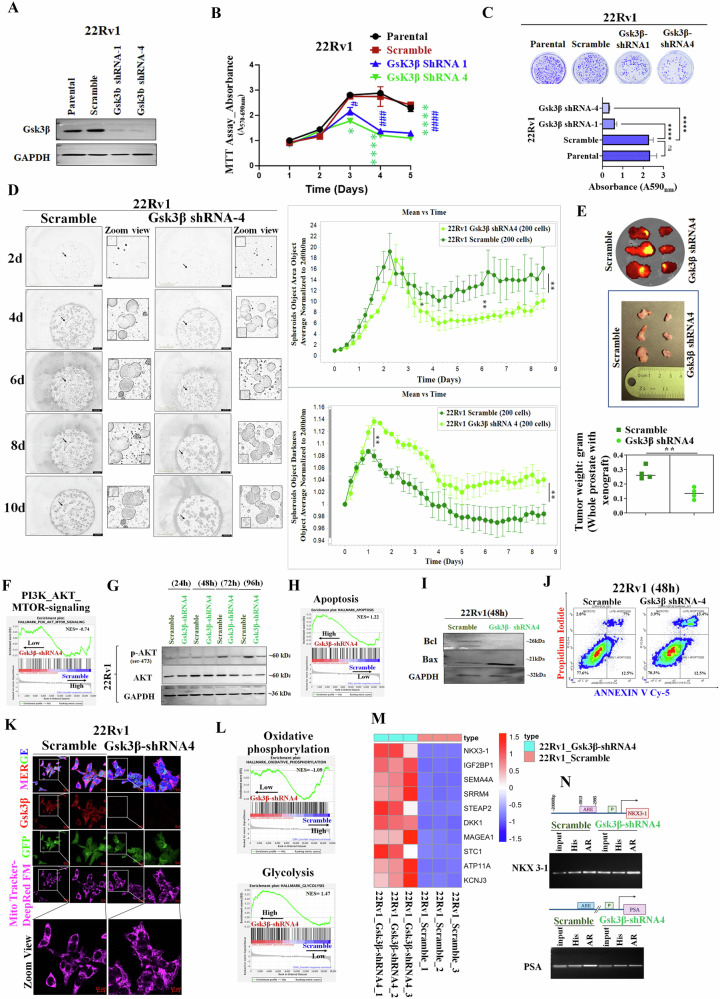
Knocking down of Gsk3β triggers an anti-proliferative response in CRPC Cells. **A** Representative WB images show the expression of Gsk3β in 22Rv1- Gsk3β KD vs. scramble cells. GAPDH was used as a loading control. **B** Cell viability assay demonstrates the growth pattern of 22Rv1- Gsk3β KD vs. scramble cells in a time-dependent manner. Values are expressed as mean ± SEM (*n* = 5). Statistical significance was defined as P ≤ 0.05. For group comparisons, *P < 0.05, **<0.01, ***<0.001 and ****P < 0.0001 for Gsk3β KD (shRNA4) vs. scramble cells; ^#^P < 0.05, ^##^P < 0.01, ^###^P < 0.001, and ^####^P < 0.0001 for Gsk3β KD (shRNA1) vs. scramble cells. **C** Evaluation of in vitro colony formation capabilities in 22Rv1- Gsk3β KD (shRNA1 and shRNA4 clone), scramble, and parental cells (upper panel). Quantitative analysis of these colonies is represented (lower panel), and values are expressed as mean ± standard error of the mean (SEM, *n* = 5).Statistical significance was considered at P ≤ 0.05. For between-group comparisons, ****P < 0.0001. **D** Representative images of the corresponding spheroids (left panel) were presented and collected with the Incucyte System. Incucyte-based real-time kinetics of 3D/matrix-multi spheroids growth presented as spheroids object area (upper panel) and spheroids darkness (lower panel) of 22Rv1- Gsk3β KD (shRNA4 clone) vs. scramble cells (right panel), One 4× image per well is taken every 6 h, and data are presented as mean ± SEM; *n* = 3. P ≤ 0.05 was considered to indicate statistical significance. **P < 0.01, *<0.05 compared with Scramble group. **E** Representative fluorescent images and picture of xenograft tumors (upper panel), quantitative analysis of tumor volume (lower panel) of scramble vs. Gsk3β KD. **F** Gene set enrichment analysis (GSEA) on RNA-seq data of Gsk3β KD vs. scramble cells. The enrichment plot represents PI3K_AKT_MTOR pathway analysis in these groups. **G** Representative WB images show the expression of p-AKT and AKT in 22Rv1- Gsk3β KD vs. scramble cells in time-dependent manners. GAPDH was used as a loading control. **H** The enrichment plot represents apoptosis pathway GSEA in Gsk3β KD vs. scramble cells. **I** Representative WB images show the expression of Bcl and Bax in 22Rv1- Gsk3β KD vs. scramble cells. GAPDH was used as a loading control. **J** Representative micrograph of apoptosis determined by flow cytometric analysis of annexin-V Cy-5/PI- dual stained cells (AV+/PI–intact cells; AV/PI+–nonviable/necrotic cells; AV+/PI and AV+/PI+–apoptotic cells) in 22Rv1- Gsk3β KD vs. scramble **K** Mitochondria fragmentation was determined by using Mitotracker Red (100 nm for 30 min) in 22Rv1- Gsk3β KD vs. scramble cells **L** Enrichment plot illustrating the GSEA of oxidative phosphorylation and glycolysis gene sets of Gsk3β KD vs. scramble cells. **M** Heatmap displays the top 10 significant upregulated genes related to RNA-seq analysis of Gsk3β KD and scramble cells. **N** ChIP assay illustrates the binding of AR to the endogenous NKX3-1 or PSA gene in Gsk3β KD vs. scramble cells.

To investigate the Gsk3β mediated generation of t-Gli3, we first generated Gsk3β knockdown (KD)- 22Rv1 cells by utilizing a human shRNA lentiviral particle system (Fig. S6A and S6B) and determined the KD efficiency (Fig. S6C, D, and 5A). To elucidate the functional impact of the reduction in Gsk3β expression, we examined the cell viability assay (Fig. 5B) and noted a significant suppression in growth on the third day. This data was further validated by analyzing real-time growth kinetics using an incucyte system (Fig. S7A). Additionally, we examined the cellular morphology through fluorescence imaging and observed distinct morphological alterations, including merged and enlarged structures, compared to the scrambled group (Fig. S7B). Subsequently, we observed fewer colonies in Gsk3β KD cells than in the scrambled control and parental cells (Fig. 5C). Using a 3D/spheroids culture system, we observed a reduction in spheroid size with Gsk3β KD (Fig. 5D). We quantified the spheroid object area (Fig. 5D upper panel) and darkness (Fig. 5D lower panel) and found a decrease in the object area along with an increase in darkness, indicating both growth suppression and enhanced dead populations. However, we did not find any phase cell cycle arrest between scramble and Gsk3β KD (Fig. S7C). The Gsk3β KD significantly decreased tumor growth and volume in the PCa xenograft mice model (Fig. 5E). Additionally, Gene Set Enrichment Analysis (GSEA) was performed using the RNA-Seq of 22Rv1-Gsk3β KD vs. scramble cells and selected altered ten major upregulated and downregulated HALLMARK GSEAs out of the top 20 for each group, which are related to oncogenic and metabolomic shift (Fig. S7D). We noticed that PI3K/AKT/mTOR signaling pathway in the top 20 enriched GSEAs in scramble cells compared to Gsk3β KD cells (Fig. 5F). This finding was substantiated by the decrease in p-AKT protein expression observed after 72 h in Gsk3β KD cells, aligning with the observed inhibitory growth kinetics in Gsk3β KD cells compared to the scramble (Fig. 5G). In summary, the outcomes derived from diverse assays collectively emphasize the pivotal role of Gsk3β in regulating the cellular proliferation of CRPC cells.

### Manifestations of apoptosis, mitochondrial dysfunction, and Metabolic reprogramming evident with Gsk3β inhibition

The GSEA analysis showed that apoptosis enriched out of the top 20 altered HALLMARK GSEAs in Gsk3β KD cells compared to scramble cells (Fig. 5H). In order to unravel the underlying apoptosis pathway, we analyzed the expression analysis of apoptotic proteins, such as Bcl, Bax, cleaved PARP, cleaved caspase-9, and cleaved caspase-3 and observed the downregulation of the anti-apoptotic Bcl protein and the upregulation of the pro-apoptotic Bax in Gsk3β KD cells at 48 h compared to scramble (Fig. 5I). Surprisingly, no significant changes in the expression of caspases were observed between the Gsk3β KD cells and the scramble condition (Fig. S7E). However, the FACS assay indicated an increased population of late apoptotic cells in Gsk3β KD cells compared to the scramble (Fig. 5J and S7F). Similarly, no substantial alterations were observed in the autophagic markers, such as Beclin-1 and SQSTM1/p62, between the Gsk3β KD cells and scramble, suggesting the involvement of pathway, independent of the caspase-regulated cell death (Fig. S7E). Considering the role of Gsk3β in mitochondrial respiration and association with apoptosis, the morphology determination of mitochondria using Mitotracker staining showed significant fragmentation of mitochondria in the Gsk3β KD cells (Fig. 5K). This finding suggests that lack of Gsk3β contributes to mitochondrial dysfunction, which is known to be associated with apoptotic cell death.

The GSEA revealed that Gsk3β KD cells displayed significant enrichment in the glycolysis pathway phenotype compared to scramble cells. Interestingly, in scramble cells, the oxidative phosphorylation phenotype was enriched in out of 20 hallmark GSEA (Fig. 5L). This metabolomic rewiring was confirmed by evaluating oxygen consumption rate/mitochondrial respiration. We observed the significant downregulation of the OCR rate (at the basal respiratory rate) in Gsk3β KD cells compared to scramble (Fig. S7G). This shift suggests that the Gsk3β might be a potential regulator for maintaining oxidative phosphorylation as an energy source for PCa cell survival.

Subsequent to this, in order to comprehend the effects of Gsk3β KD at the gene level, we ran differential expression analysis (DEA) to identify the top 10 up and down-regulated genes in Gsk3β KD vs. scrambled cells. Our DEA highlighted that NKX3-1 is the top upregulated gene (Fig. 5M). NKX3-1, a prostate-specific tumor suppressor gene, is transcriptionally regulated by AR and considered the downstream target of Shh in the prostate [28]. It operates by stabilizing P53 and inhibiting AKT activation, thus suppressing the initiation of PCa [29]. In addition, NKX3-1 was. In our gene set enrichment analysis, we observed the upregulation of P53 and the downregulation of the AKT pathway (Fig. S7D). To further corroborate these findings, we conducted chromatin immunoprecipitation (ChIP) assays to investigate the binding of AR to transcriptionally active genes, including NKX3-1 and the well-known PSA (Fig. 5N) In Gsk3β KD cells compared to scramble cells, we noted an augmentation in AR binding to NKX3-1, whereas there was a reduction in AR binding to PSA genes. However, further investigation is needed to examine whether the upregulation of NKX3-1 acts as a potential regulator in the PCa growth inhibition by Gsk3β KD, as suggested by the data.

### Potential involvement of Gsk3β in the generation of t-Gli3 and facilitating interaction between t-Gli3 and AR-V7

**Fig. 6:**
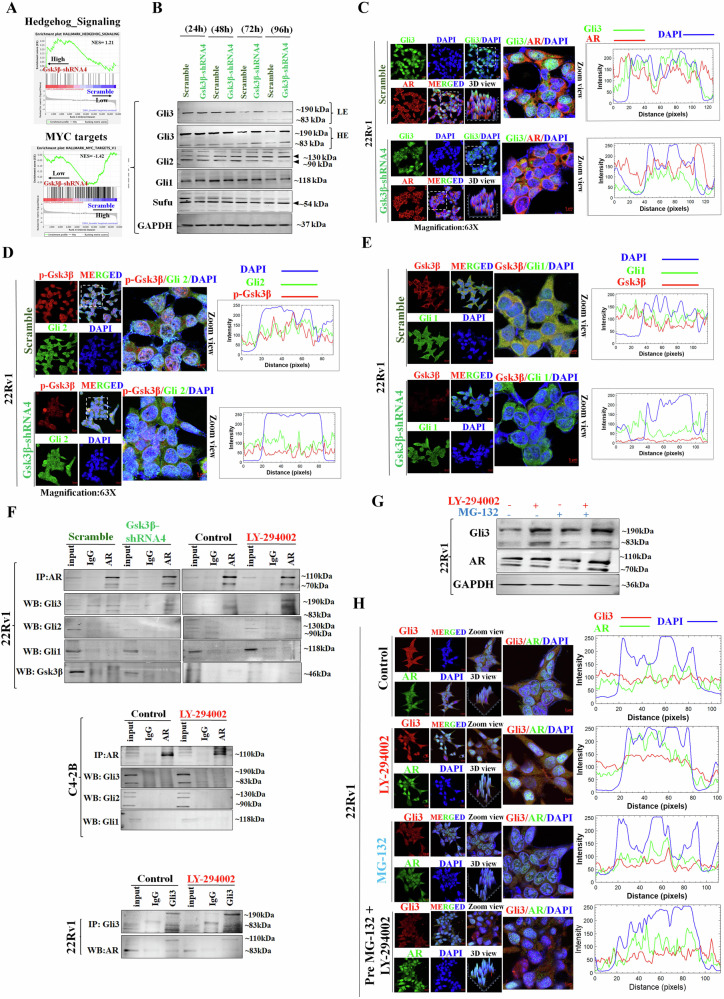
Impact of Gsk3β knockdown on Hh/Gli cascade and processing of Gli3. **A** Enrichment plot illustrating the GSEA analysis for the Hh pathway and MYC targets in Gsk3β KD cells compared to scramble cells. **B** Representative WB images show the expression of Gsk3β, Gli proteins, and Sufu in 22Rv1- Gsk3β KD vs. scramble cells for different time points. **C**–**E** Representative IF images (left panel) and fluorescence intensity plot (right panel) of co-expression of Gli3/AR, p-Gsk3β/Gli2, and Gsk3β/Gli1 in 22Rv1- Gsk3β KD vs. scramble cells. **F** Representative WB images show the expression of AR and Gli proteins following IP with AR in 22Rv1- Gsk3β KD vs. scramble cells (left panel), 22Rv1 cells treated with Gsk3β activator vs. untreated (control) cells (right panel). AR and Gli proteins were expressed following IP with AR in C4-2B cells treated with Gsk3β activator vs. untreated (control) cells (middle panel). Gli3 and AR proteins were expressed following IP with Gli3 in 22Rv1 cells treated with Gsk3β activator vs. untreated (control) cells (lower panel). **G** Representative WB images show the expression of Gli3 and AR in different treated groups. GAPDH was used as a loading control. **H** Representative IF images (left panel) and fluorescence intensity plot (right panel) of co-expression of Gli3/AR in indicated cells.

Examining the Gsk3β-mediated signaling pathway regulation, we noted an enrichment of Hh signaling in Gsk3β KD cells and a significant enrichment of MYC targets in scramble cells (Fig. 6A). This observation further reinforces the notion that Gsk3β serves as a master regulator of the Hh pathway. Subsequently, we examined the effect of Gsk3β KD on Gli proteins and noted that the expression of Gli1, Gli2, and Sufu remained unchanged. However, a noteworthy upregulation was observed in the Gli3 expression, particularly the FL-Gli3, at both 72 h and 96 h (Fig. 6B). The observed increase in FL-Gli3 suggests that inhibition of Gsk3β may influence the processing of Gli3, potentially inhibiting the generation of t-Gli3. Furthermore, we performed co-expression analyses of Gli3/AR, Gli2/p-Gsk3β, and Gli1/Gsk3β using confocal microscopy (Fig. 6C–E) and fluorescence intensity was analyzed. We did notice a loss of nuclear expression of Gli3 in Gsk3β KD cells, indicating that inhibition of Gsk3β impedes the nuclear translocation of Gli3. Similar to the WB results, we did not observe any changes in the expression levels of Gli1 and Gli2 between Gsk3β KD and scramble cells. These observations suggest a potential regulatory role of Gsk3β for the nuclear translocation of t-Gli3 and subsequent modulation of Gli3-mediated signaling response. Taken together, gene set enrichment and protein expression analysis, we can again conclude that the presence of Gsk3β regulates Gli cascade at the protein level of Gli3 processing.

To substantiate the earlier observations regarding the reliance of t-Gli3 generation on the presence of Gsk3β and its specific interaction with AR-V7, we conducted experiments involving Gsk3β inhibition and Gsk3β activation in PCa cells through IP (Fig. 6F). In our investigation with Gsk3β KD-22Rv1 cells expressing both AR-FL and AR-V7, the reduction in t-Gli3 expression, compared to the scramble following AR enrichment, indicated a lack of interaction between AR and Gli-family proteins (Gli1, Gli2, and t-Gli3) after Gsk3β knockdown (Fig. 6F left panel). Conversely, upon Gsk3β activation using LY-294002 in 22Rv1 cells, a significant increase in both FL-Gli3 and t-Gli3 expression was observed, suggesting the facilitation of the interaction between Gli3 and AR in the presence of Gsk3β (Fig. 6F right panel). Furthermore, in C4-2B cells, which only express AR-FL, we did not observe any interaction between AR and Gli-family proteins (Fig. 6F middle panel). Moreover, in reverse co-IP experiments conducted in 22Rv1 cells with Gli3 enrichment, we noticed an interaction between Gli3 and AR (Fig. 6F lower panel).

Phosphorylated-Gli3 processing is triggered in a ubiquitin-dependent manner via the SCF-βTrCP1 complex [30]. To further corroborate these crucial findings, we employed the proteasome inhibitor MG132, Gsk3β activator, or a combination of both (Fig. 6G). When the Gsk3β activator was used alone, we observed an increase in the expression of both F-Gli3 and t-Gli3. Conversely, treatment with MG132 alone resulted in the inhibition of t-Gli3 formation. Interestingly, when the group pre-treated with MG132 was subsequently treated with the Gsk3β activator, we found a significant induction of t-Gli3 expression compared to the group treated with MG132 alone. We also examined the expression of AR in these experimental conditions and found that treatment with MG132 alone was able to inhibit AR-V7 expression. However, no significant changes in AR expression were observed in the groups treated with the Gsk3β activator alone or in combination with MG132. This suggests that Gsk3β-mediated processing is specific to Gli3 and does not affect AR expression. Indeed, the additional confocal imaging analysis allowed us to visually confirm the expression pattern of Gli3 and AR (Fig. 6H). The consistent results obtained from confocal microscopy and WB provide robust evidence supporting the conclusion that Gsk3β activator can override the inhibitory effect of MG132 on t-Gli3 formation, leading to an increase in t-Gli3 expression.

### Concurrent inhibition of Gsk3β along with Smo or Gli1 leads to an improved cytotoxic effect in CRPC cells

**Fig. 7:**
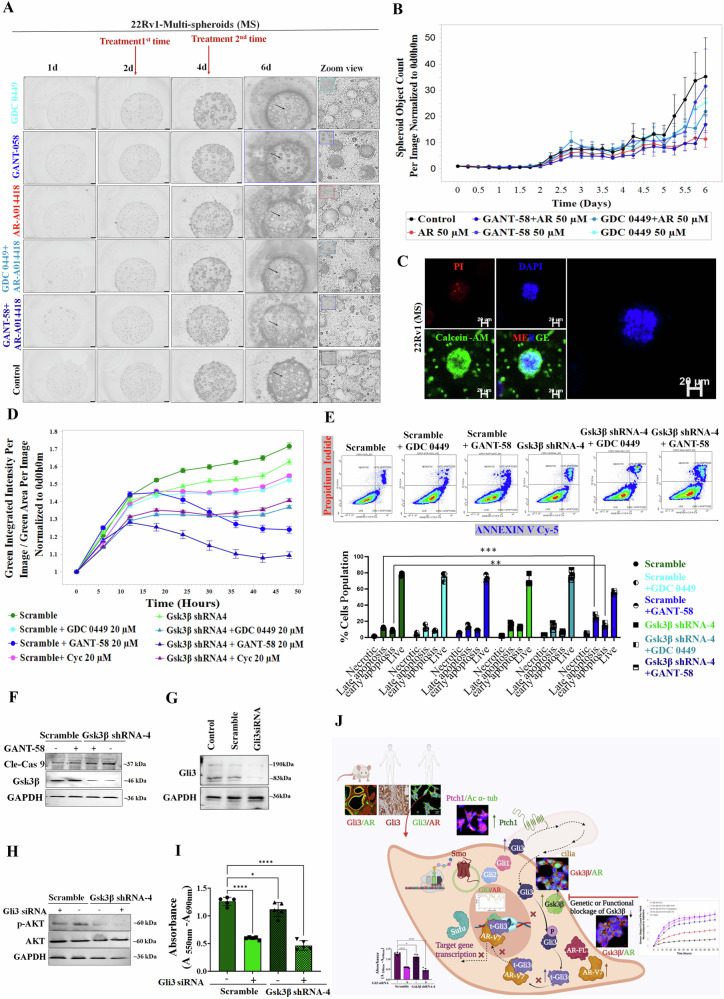
Cytotoxic effects of concurrent Gsk3β inhibition with either Smo or Gli1 or solely Gli3 inhibition. **A** Representative images of the corresponding spheroids of 22Rv1 cells treated with different inhibitors as indicated were presented, which were collected with the Incucyte System. **B** Incucyte-based real-time kinetics of 3D/matrix-multi spheroids growth (presented as spheroids object area) of 2Rv1 cells treated with different inhibitors, One 4× image per well is taken every 6 h, and data are presented as mean ± SEM; *n* = 3. **C** Representative morphology of control spheroids of 22Rv1 stained with Calcein AM and PI. **D** Incucyte-based real-time growth kinetics of Gsk3β KD vs. scramble cells treated with different inhibitors, One 4× image per well is taken every 6 h, and data are presented as mean ± SEM; *n* = 3. **E** Representative micrograph of apoptosis assessed through flow cytometric analysis of annexin-V Cy-5/PI- dual stained cells (AV+/PI–intact cells; AV/PI+–nonviable/necrotic cells; AV+/PI and AV+/PI+—apoptotic cells) in 22Rv1- Gsk3β KD and scramble cells treated with Smo (GDC-0449) or Gli1(GANT-58) inhibitors (upper panel). Quantitative analysis of these micrographs was shown as mean ± SEM (*n* = 3) (lower panel).P ≤ 0.05 was considered to indicate statistical significance. **P<0.01, <***P <0.001 compared to Scramble group. **F** Representative WB images show the expression of cleaved caspase-9 in 22Rv1- Gsk3β KD and scramble cells- treated with Gli1 inhibitor (GANT-58). GAPDH was used as a loading control. **G** Representative WB images show the expression of Gli3 in 22Rv1 cells that were transfected with Gli3-siRNA. GAPDH was used as a loading control. **H** Representative WB images show the expression of p-AKT and AKT in Gsk3β KD and scramble cells; both were transfected with Gli3-siRNA as indicated. GAPDH was used as a loading control. **I** Cell viability (MTT assay) of Gsk3β KD and scramble cells transfected with Gli3-siRNA. P ≤ 0.05 was considered to indicate statistical significance. *P <0.05, ****P < 0.0001 compared with Scramble group. **J** Schematic representation of proposed hypothesis.

We investigated the therapeutic potential of Smo/Gli cascade inhibition using Cyclopamine and its analog, Tomatidine. However, no cytotoxic effects were observed with these inhibitors (Fig. S8A). While Cyclopamine effectively downregulated Smo (48h, 72 h) and Gli1 (72h) over time (Fig. S8B), it did not significantly impact the cell viability in CRPC cells under serum starvation and androgen deprivation (Fig. S8C). However, preincubation with DTH led to growth inhibition upon Cyclopamine treatment (Fig. S8D). We assessed additional Hh inhibitors, GDC0149 (a Smo inhibitor) and GANT-58 (a Gli1 inhibitor), on PCa cell growth kinetics. Despite effectively targeting/modulating Smo and Gli1 expression (Fig. S8E), these inhibitors did not significantly impact cell viability and apoptosis (Fig. S5F, and S5G, H). In contrast to limited efficacy when inhibiting Smo or Gli1 alone, the combination of Smo/Gli1 and Gsk3β inhibitors demonstrated significant efficacy in inhibiting cellular viability (Fig. S9A,B). Following the intriguing results, we extended our investigation by employing a 3D culture system (spheroid assay). Similarly, in a 3D spheroid assay, the combination of GDC-0449 and AR-A014418, as well as GANT-58 and AR-A014418, resulted in reduced spheroid count compared to individual or untreated groups (Fig. 7A, B). To confirm the successful formation of spheroids using 22Rv1 cells, we performed fluorescence imaging utilizing Calcein AM and PI staining (Fig. 7C). These findings shed light on the potential synergistic effects of simultaneously targeting Gsk3β along with Smo or Gli1 in inhibiting cell viability, indicating a promising therapeutic approach.

Furthermore, the growth inhibitory effects of Hh inhibitors (Cyclopamine, GDC-0449, and GANT-58) on Gsk3β KD CRPC cells were observed as time-dependent (Fig. S9C and 7D). Additionally, an apoptosis assay using FACS revealed a significant increase in the apoptotic population in GANT-58-treated Gsk3β KD cells compared to the other groups (Fig. 7E). This was further supported by notable upregulation of cleaved caspase-9, indicating apoptotic pathway activation in GANT-58 treated Gsk3β KD cells (Fig. 7F).

### Inhibition of Gli3 significantly suppresses the growth of CRPC cells

To examine the direct effect of Gli3 inhibition on the survival of CRPC cells, we used Gli3 small interfering RNA (siRNA) and observed an approximate 80% downregulation of Gli3 expression in both isoforms (Fig. 7G). Next, p-AKT expression levels were significantly downregulated in the Gli3siRNA-treated groups, providing further evidence that Gli3 inhibition effectively inhibits cell growth (Fig. 7H). Furthermore, MTT assays conducted on Gsk3β KD cells treated with Gli3siRNA revealed a significant reduction in cell growth, particularly in the Gli3siRNA-treated Gsk3β KD cells. However, significant inhibitory effect was also observed in the scramble group (Fig. 7I). These results indicate that Gli3 inhibition significantly impacts the survival of CRPC cells, both in the context of Gsk3β knockdown and independently, highlighting its efficiency in inhibiting cell growth.

## Discussion

The oncogenic effects of the Hh signaling rely on the activity of Gli transcription factors, which are regulated through either canonical receptor-mediated or non-canonical mechanisms. Both modes of regulation lead to modifications to the post-translational processing of Gli proteins, ultimately influencing their activity and downstream effects in cellular processes.

In our study, human clinical samples, MYC^OE^ prostatic mouse tissue, and CRPC human cells showed increased Gli3 expression with predominantly nuclear accumulation. MYC^OE^ PCa models are advantageous as they represent benign, early, and late-stage diseases according to gene dosage and/or latency. Additionally, oncogene c-MYC regulates Gli1 expression independently of Smo, Ptch, or the presence of Hh ligands in Burkitt lymphoma [31]. Therefore, we used this model to prove our hypothesis and determine the expression of Gli3. However, the Gli1, Gli2, and Gli3 proteins showed distinct sub-cellular localization in clinical samples as well as CRPC cells. For in-depth analysis, we utilized a diverse set of human PCa cell lines based on the presence of the AR and their reliance on AR signaling along with exogenous exposure of AR inhibitor and activator. Interestingly, we observed Gli1 and Gli2 expression decreased, while Gli3, especially t-Gli3, increased in PCa cell lines compared to normal prostate cells. The regulatory component of Gli proteins, Sufu, remained unchanged. These findings imply that Gli3 may have a potential role in the progression of advanced PCa independently of Sufu. In addition, AI/CRPC cell lines showed higher Gli3 expression compared to AD and normal cells. These findings substantiated the role of Gli3 in the progression of CRPC through its involvement with AR pathways. Notably, we observed that the ligand-dependent Shh/Gli axis of the canonical pathway is involved in response to serum starvation. In contrast, the non-canonical pathway, characterized by ligand-independent signaling, is implicated under ADT.

Next, by unraveling the canonical regulation of Gli3, which is typically influenced by receptor-mediated ciliary localization [32, 33], we noted some interesting observations. The absence of ciliary localization of the Smo receptor and the presence of the Ptch receptor implied the existence of a Smo-independent action that supported the non-canonical mechanism for the upregulation of Gli3. As both Smo and Ptch proteins act as receptors for Gli activation and play a crucial role in mediating its action [15]. Ptch is also considered a transcriptional downstream target of the Hh/Gli cascade [34]. Despite the absence of experimental evidence identifying potential downstream targets of Gli3 [33], the increased Ptch1 and decreased Smo expression in CRPC cells further support Gli3’s role in advanced PCa progression. Traditionally, the Hh/Gli axis is associated with the role of Gli1 and Gli2 as activators, while Gli3 acts as a suppressor [14, 15]. However, this study suggests that Gli3 may function as an activator for the Hh pathway in the presence of AR, potentially influenced by non-canonical/non-classical mechanisms.

A study revealed that the AR interacts with Gli3, hindering βTrCP from binding to Gli3. As a result, Gli3 is not processed into its repressor form, leading to the non-canonical activation of the Hh signaling pathway in PCa cells [23]. Additionally, when AR was knocked down, an increase in the repressor form of Gli3 was observed. These findings emphasize that the presence of AR suppresses the generation of repressor form and promotes the binding with FL-Gli3. Furthermore, independent of androgen, a variant of LNCaP cells exhibited a higher endogenous ratio of Gli3-FL/Gli3R [23]. Contrary to this, we noted that most PCa cell lines predominantly expressed the repressor form of Gli3, referred to as t-Gli3 (Fig. 1F). However, few cell lines showed abundant expression of both forms of Gli3 proteins, indicating heterogeneity in Gli3 expression in PCa. This prompted us to investigate the role of t-Gli3 in PCa cell growth, its interaction with AR, and the mechanisms responsible for its generation. This novelty, particularly the significance of t-Gli3 as a focal point, highlights a previously unexplored area within the field. Regarding AR-mediated action, we observed a significant positive correlation between Gli3 and AR, with no correlation for Gli1 or Gli2 in PCa patients. Understanding how t-Gli3, lacking a transactivation domain, instills the non-canonical Gli cascade is an important area of investigation. It is well documented that AR functions as a binding partner for numerous proteins; hence, we performed a protein-protein interaction assay in LNCaP-135 and 22Rv1 cells which express both AR-FL and AR-V7, and revealed that the presence of AR-V7 is sufficient for t-Gli3 activity. This finding was reinforced by the absence of an interaction between t-Gli3 and AR in C4-2B cells, which lack AR-V7. These observations highlighted the crucial role of a selective interaction between t-Gli3 and AR-V7 in facilitating the function of t-Gli3. AR-V7 is derived from AR-FL splicing and retains the complete N-terminal domain (NTD) and DNA-binding domain (DBD) but lacks the ligand-binding domain (LBD) and hinge domain (HD), which are replaced by a peptide from cryptic exon 3 (CE3). This structure enables AR-V7 to have a complete activation function (AF)-1 transactivation domain, making it constitutively active and capable of maintaining gene transcription without androgen binding, thereby contributing to ENZ resistance [35, 36]. With this background, we made an intriguing observation of t-Gli3 binding to AR-V7 in PCa cell lines that express both AR-FL and AR-V7, rather than those that express only AR-FL. This important observation warrants further exploration using knockout strategies to remove either AR-FL or AR-V7, individually or in combination, in PCa cells.

Recognizing the association of AR-V7 with t-Gli3 and its role in Gli3-mediated activity, we investigated factors involved in Gli3 processing. The biochemical structure of FL-Gli3 contains a Gsk3β binding site, emphasizing Gsk3β‘s role in its processing. We found increased Gsk3β expression in primary prostate tumors and a positive correlation with AR. Additionally, we noted distinct expression patterns of Gsk3β and p-Gsk3β in PCa cell lines based on AR status, with elevated Gsk3β in AI cell lines compared to AD or normal prostate cells. Remarkably, both human TMA analysis of PCa patients and PCa cell lines represented increased cytosolic and nuclear localization of Gsk3β in CRPC cells, underscoring its importance in CRPC.

Furthermore, Based on these findings, in our study we used a selective Gsk3β activator (LY-294002) [17, 37] and an inhibitor (AR-A014418) [38]. Inhibiting Gsk3β led to a significant reduction in cell survival, indicating its importance in promoting cell survival. Conversely, activating Gsk3β did not show a noticeable effect on cell survival. Understanding the Gsk3β/Gli3 axis, upon treating cells with the Gsk3β activator, Gsk3β predominantly translocated to the nucleus, leading to increased expression of Gli3, particularly t-Gli3, at 2 h, and both form at 6 h, indicating that Gsk3β activation affects Gli3 expression and processing.

However, few reports considered AR-A014418 as a selective Gsk3 inhibitor and mediated its action by a reduction in phosphorylation of Gsk3α [39, 40]. Hence, we investigated the functional consequences of reducing specifically Gsk3β expression through Gsk3β shRNA using 2D and 3D culture platforms of 22Rv1 cells. Our findings revealed significant growth suppression on the third day, which became more pronounced with reduced colony formation and decreased p-Akt expression. This suggests inhibition of the cell survival and proliferation pathway, with Akt serving as a key mediator [41]. Moreover, in vivo, orthotropic implantation of Gsk3β KD cells in mice showed decreased tumor volume compared to scramble, highlighting the critical role of Gsk3β as an oncogenic factor. Mechanistically, we observed mitochondrial fragmentation and changes in apoptotic markers Bcl and Bax in Gsk3β KD cells, with no changes in caspase expression, indicating apoptosis occurred through alternative pathways independent of caspases or autophagy.

Exploring the key pathways affected by Gsk3β, GSEA HALLMARK analysis of RNA-seq data from Gsk3β KD versus scramble cells revealed that Gsk3β blockage induced a metabolic reprogramming shift toward glycolysis, stimulated apoptosis, activated tumor suppressor genes, and inhibited oncogenic signaling in Gsk3β KD cells. Markedly, normal prostatic epithelium employs comparatively glycolytic metabolism to sustain physiological citrate secretion. PCa has a unique metabolism characterized by an intact tricarboxylic acid cycle. Prostate adenocarcinoma consumes citrate to power oxidative phosphorylation and fuel lipogenesis to sustain cellular proliferation, i.e., different from other cancer metabolic profiles [42]. In addition, AR signaling controls PCa metabolism. Hence, this shift suggests the potential role of Gsk3β for maintaining oxidative phosphorylation as an energy source for PCa cell survival.

Among the top 10 up-and down-regulated gene expressions from RNA-seq analysis, we have validated the role of the upregulated NKX3-1 gene, a prostate-specific tumor suppressor, which was the top hit. *NKX3-1* is considered a downstream of Shh, and a recent study investigated a mechanism of Gsk3β-dependent degradation NKX3-1 [28, 43]. In addition, a recent study showed that loss of NKX3-1 is associated with hormone-refractory disease and advanced tumor stages in PCa [44]. Notably, our ChIP analysis demonstrated significant AR binding to the ARE of NKX3-1, in Gsk3β KD cells. Recent research revealed that restoration of NKX3-1 contributes to stabilizing P53, inhibiting AKT activation, and preventing PCa initiation caused by PTEN loss by inhibiting cell proliferation and inducing apoptosis [29]. Similarly, our GSEA indicated modulation of the P53 and AKT pathways, suggesting that Gsk3β KD-mediated upregulation of NKX3-1 may contribute to the regulation of these pathways. Moreover, concerning the Gsk3β/Gli3 axis, we observed a Hh pathway gene enrichment and a significant increase in the protein expression of Gli3, specifically the Gli3-FL in Gsk3β KD cells cultured for 72 and 96 h. Furthermore, a decrease in the nuclear localization of Gli3 indicates that inhibiting Gsk3β hinders the translocation of Gli3 into the nucleus. However, the expression levels of other regulatory components, such as Gli1, Gli2, Sufu, and AR, remained unchanged in these cells. These findings suggest that inhibiting Gsk3β activity reduces the production of t-Gli3, resulting in decreased nuclear accumulation of Gli3. Based on these findings, we can conclude that the reduced activity of Gsk3β inhibits the production of t-Gli3, leading to a decrease in the nuclear accumulation of Gli3. These findings suggest the significance of Gsk3β in Gli3 processing and its role in generating the t-Gli3. Fascinatingly, we found no interaction between Gli3 and AR was detected in Gsk3β KD, but Gsk3β activation significantly enhanced this interaction in 22Rv1 cells, expressing AR-FL and AR-V7. Interestingly, no significant interaction was observed in C4-2B cells that primarily express AR-FL, even after Gsk3β activation. These findings suggest that the presence of the AR-V7 variant may influence the Gli3-AR interaction upon Gsk3β activation. Sequential phosphorylation in the binding induction of Gli3 and βTrCP1 is a hallmark to authenticate Gli3 proteolytic processing [30]. We also confirmed that Gsk3β specifically mediates Gli3 processing without affecting AR, using the proteasome inhibitor MG132.

Furthermore, we assessed the therapeutic impact of targeting Hh signaling components in various PCa cell lines (androgen-dependent and -independent) using 2D and 3D/spheroid models. The combined Smo or Gli1 inhibition approach with Gsk3β blockage, either through an inhibitor or knockdown, showed synergistic cytotoxic effects compared to targeting Smo or Gli1 alone in CRPC cells. In addition, Gsk3β KD cells treated with a Gli1 inhibitor showed greater potential and mechanistically demonstrated increased apoptosis compared to other treatments. These findings shed light on the limited efficacy of Smo or Gli1 inhibitors in clinical trials [11, 12].

Additionally, inhibiting Gli3 (FL- Gli3 and t-Gli3) with Gli3siRNA significantly decreased the survival of parental 22Rv1 cells, with a more pronounced effect in Gsk3β KD-22Rv1 cells. These findings align with a previous study that reported Gli3 KD suppressed castration-resistant cells and tumor growth [24]. Our study underscores the importance of t-Gli3 in PCa cell growth, resistance, and cellular responses, enhancing our understanding of Gli3’s multifaceted role in androgen response and PCa biology.

In summary, our study advances the comprehensive understanding of the Gli-family proteins Gli1, Gli2, and Gli3, likely “one-size fits all” and explores non-canonical Gli3 response in PCa biology. A distinctive aspect of this study is the recognition of the significance of t-Gli3 as an activator, facilitated by its interaction with AR-V7, independently of Smo and Sufu. These insights highlight the crucial role played by increased Gsk3β activity in post-translational modification, leading to the generation of t-Gli3. This truncated proteoform interacts with AR-V7, translocating to the nucleus to activate AR or Hh target genes (Fig. 7J). The comprehensive analysis of the global pathways and genes in the context of the genetic blockade of Gsk3β revealed the modulation of crucial oncogenic and apoptotic pathways, accompanied by the upregulation of the NKX3-1 gene. Combining Gsk3β inhibition with Hh signaling components significantly inhibited growth, highlighting Gsk3β‘s role in non-canonical Hh/Gli signaling. Importantly, solely targeting Gli3 effectively inhibited CRPC cell growth, underscoring its potential as a promising therapeutic target in PCa.

Our future research will focus on the oncogenic role and therapeutic potential of t-Gli3 through selective deletion or overexpression and its post-translational mechanisms. Exploring the fundamental intrinsic processes that generate and transport t-Gli3 in CRPC and assessing its effects on stemness, metastasis, and resistance to therapies will be a key area of investigation. Additionally, generating Gli3-overexpressing GEMM models will offer clearer insights into the significance of Gli3 in prostate cancer biology. This stage will provide valuable insights into the Gsk3β/Gli3 cascade, guiding future clinical studies and advancing our understanding of its therapeutic potential for treating PCa/CRPC patients.

## Materials and methods

### Cell lines and culture

All human PCa cell lines LNCaP LN3, LNCaP-35 (c-33), LNCaP-135 (c-81), PC3, PC3M, C4-2, C4-2B, 22Rv1, DU145, and normal prostate cell RWPE1 were tested for mycoplasma contamination and further validated by short tandem repeat (STR) DNA profiling University of Nebraska Medical Center. PCa cell lines were cultured in RPMI-1640 (Invitrogen Cat# 11875) or in Dulbecco’s Modified Eagle Medium (DMEM) media (Cytiva Cat#SH30022.01). All media were supplemented with 5% fetal bovine serum (FBS; R&D Systems Cat#S11550H) and 1% penicillin-streptomycin (Invitrogen Cat#15140-122). Cells were incubated in a humidified incubator at 37 °C and supplied with 5% CO_2_. Cells were subcultured by trypsin-EDTA treatment. We also utilized syngeneic cell lines derived from PTEN conditional knockout mice. These cells were generated from two distinct prostate tumors: primary (androgen-dependent) tumors and post-castration recurrent (androgen-depletion-independent) tumors, specifically including androgen-dependent E2 and androgen-depletion-independent cE2 cells. Both mouse prostate-derived cells were maintained in DMEM and validated for functional analyses, as described previously [45, 46].

In the in vitro experiments, cells were treated with inhibitors and/or activators and processed for assays according to the experimental requirements. The cells were exposed to charcoal-stripped fetal bovine serum (FBS) to emulate an androgen-deprived environment and serum-free media (SFM) to simulate a starved/stressed condition.

All cell culture reagents, antibodies, and chemicals used in this study are listed in the Supplementary Table.

### 3-D culture/multi-spheroid culture

For spheroids formation, we followed the published protocol with modifications [47]. 22Rv1 cells were cultured in a 24-well attachment plate, with 200 cells per well, to promote sphere formation. The culture medium RPMI was used, supplemented with specific constituents: 25 ng/ml cholexatoxin, 10 ng/ml mouse/EGF (epidermal growth factor), basic 10 ng/mL FGF (fibroblast growth factor), 0.005 mM phosphoethanolamine, 100 pg/ml hydrocortisone, and 45 ng/ml human recombinant insulin. Additionally, a hydrogel matrix, Matrigel, was used during the seeding of cells. Sphere formation and experiments were monitored for 6–7 days using the Incucyte Live-Cell Imaging analysis system (Essen Bioscience). Inhibitors were applied as needed during the study.

### HI-MYC mouse PCa model

Hi-Myc (FVB background) mice were received from the NCI Mouse Models of Human Cancers Consortium (MMHCC) in Frederick, MD, USA. The animals were allowed with food and water ad and kept under a 12-hour dark/light cycle. All animal experiments were conducted following U.S. Public Health Service guidelines for the care and use of laboratory animals, adhering to ARRIVE guidelines and approved by the UNMC Institutional Animal Care and Use Committee (IACUC). We screened male mice genotypically positive for the human MYC transgene, along with littermate controls, and followed the instructions described in our earlier published research article [48]. After sacrificing the mice, their prostate tissues were fixed in 10% neutral-buffered formalin and processed into paraffin blocks for standard hematoxylin, eosin, and IHC staining. Pathologist, Dr Lele screened the tumor stage (HGPIN or Carcinoma) and evaluated Gli3 protein staining intensity.

### In vivo xenograft study

Male athymic nude mice aged 6 weeks were used for the study. Animal experiments were performed according to the protocol approved by the Institutional Animal Care and Use Committee of the University of Nebraska Medical Center, Omaha, Nebraska.

Immunocompromised male athymic nude mice were anesthetized with ketamine/xylazine, and a 1–1.5 cm incision was made in the lower midline abdomen to implant fluorescence expressing human PCa 22Rv1-scramble/or Gsk3β cells (0.5 × 10^6^ cells/25 µl with 25 µl of Matrigel = total volume 50 µl) in the dorsal lobes of the prostate. The incision was closed with 9 wound clips, and analgesic buprenorphine was administered for 48 h post-surgery. Wound clips were removed after 7–10 days, and tumor growth was monitored weekly using IVIS spectrum imaging. Euthanasia was performed 8 weeks post-implantation in accordance with IACUC guidelines. Tumors were resected, and tumor weight was measured, as described previously [46].

### RNA-sequencing

22Rv1-Gsk3β shRNA4 or 22Rv1-scramble cells were lysed using RNA lysis buffer provided with the mirVana RNA isolation kit (Cat#AM1561, Thermo Fischer Scientific; *n* = 3). Total RNA was isolated by following the manufacturer’s protocol. A whole transcriptome analysis (RNA-sequencing) was performed on 22Rv1-Gsk3β shRNA4 and 22Rv1-scramble cells at the Sequencing Core Facility, University of Nebraska Medical Center. RNA quality was checked using Agilent Bioanalyzer (Agilent Technologies, Inc, Santa Clara, CA), and all RINs (RNA integrity numbers) were 10. Library preparation was achieved using the Illumina TruSeq RNA Sample Preparation Kit according to the manufacturer’s protocol. Library preparation, PCR amplification, size distribution, library quantification, and sample loading were performed as described previously [49]. Sequencing was performed in rapid mode on a HiSeq2500 sequencer (Illumina Inc., San Diego, CA). A single read, 50 cycles, sequencing run onboard clustering, and V2 chemistry were used.

### Bioinformatics analysis

The OncoDB [50] was used for all the correlation analyses. The mRNA expression of Gli-family transcription factors and Gsk3β kinase were analyzed using the Cancer Genome Atlas (TCGA) PRAD and GTEx database. The raw sequences of RNA-seq were processed for quality control using both FastQC [51] and MultiQC [52]. Alignment was done using HISAT2 [53] after checking the quality of the reads. The GRCh38 release 84 version of H. sapiens (human) from Ensembl was used as a genome reference. Next, expression quantification was done in R using “featureCounts” [54] from the “Rsubread” package. Differential expression analysis was run in R using the DESeq2 package [55]. Gene Set Enrichment Analysis was run using GSEA version 4.3.2 [56]. Both heatmap and volcano plot were generated in R using ggplot2.

### Quantification and statistical analysis

Statistical analysis and graphical representations were performed using GraphPad Prism 8, except for real-time growth kinetic assays for cells and spheroids, where the IncuCyte software was used to generate graphs. Data are presented as mean ± standard deviation (SD) or mean ± SEM. Significance was determined using a simple student *t-*test or multiple comparisons. A (*p*-value < 0.05) was considered statistically significant. We used a *p*-value < 0.05 for correlation analysis, which was considered statistically significant. A positive correlation presents a weak positive correlation. If the *r* value is between 0.25 and 0.5 and the *r* value is between 0.5 and 0.7, it is considered a moderate positive correlation, and if the *r* value is >0.7, it is considered a strong positive correlation. For confocal colocalization analysis, if the Pearson coefficient *r* value is >0.6, it is considered a strong correlation.

All the in vitro experiments were repeated three times with biological and technical replicates. Further statistical details can be found in the figure legends.

Furthermore, all other experimental methods used in this study are available in the Supplementary Materials and Methods file.

## Supplementary information

Supplementary Figures

Supplementary Table

Supplementary Material and Methods

## Data Availability

RNA-sequencing data have been deposited to Gene Expression Omnibus (GEO) with an accession: GSE255648. Any additional information related to this manuscript is available from the lead contact upon request.
