# Supplementary material for: Oncogenic potential of truncated-Gli3 via the Gsk3β/Gli3/AR-V7 axis in castration-resistant prostate cancer
Source: Oncogene. 2025 Jan 16;44(15):1007–23. doi: 10.1038/s41388-024-03266-z (PMC11976299; doi:10.1038/s41388-024-03266-z)
Supplement: Supplementary file 1 — Supplementary Figures [file 41388_2024_3266_MOESM1_ESM.docx]

**SUPPLEMENTARY FIGURES**

**Oncogenic Potential of Truncated-Gli3 via the Gsk3β/Gli3/AR-V7 Axis in Castration-Resistant Prostate Cancer**

Jyoti B Kaushal^1*^, Pratima Raut^1^, Sushanta Halder^1^, Zahraa W Alsafwani^1^, Seema Parte^1^, Gunjan Sharma^1,5^, K M Abdullah^1,5^, Parthasarathy Seshacharyulu^1^, Subodh M Lele^4^, Surinder K Batra^1,2,3, *,^ Jawed A Siddiqui^1,2,5.6 *^

*^1^Department of Biochemistry and Molecular Biology, University of Nebraska Medical Center, Omaha, NE USA.*

*^2^Fred and Pamela Buffett Cancer Center, University of Nebraska Medical Center, Omaha, NE, USA.*

*^3^Eppley Institute for Cancer and Allied Diseases Research, University of Nebraska Medical Center, Omaha, NE, USA.*

*^4^Department of Pathology and Microbiology, University of Nebraska Medical Center, Omaha, NE, USA*

*^5^Department of Cell and Molecular Biology, University of Mississippi Medical Center, Jackson, MS, USA*

*^6^Cancer Center Research Institute, University of Mississippi Medical Center, Jackson, MS, USA*

*Running title: role of t-Gli3 and Gsk3β-mediated Gli3 processing in CRPC*

*Corresponding author:

Jyoti B Kaushal, Ph.D., Surinder K Batra, Ph.D. & Jawed A Siddiqui, Ph.D.

For correspondence: Jyoti B Kaushal, Ph.D. Department of Biochemistry and Molecular Biology, University of Nebraska Medical Center, Omaha, Nebraska, 68198-5870, USA. Email: [jyoti.kaushal@unmc.edu](mailto:jyoti.kaushal@unmc.edu)

Surinder K. Batra, Ph.D. Department of Biochemistry and Molecular Biology, University of Nebraska Medical Center, Omaha, Nebraska, 68198-5870, USA. Phone: 402-559-5455, Fax: 402-559-6650, Email: [sbatra@unmc.edu](mailto:sbatra@unmc.edu)

Jawed A Siddiqui, Ph.D. Department of Cell and Molecular Biology, University of Mississippi Medical Center, Jackson, Mississippi, 39216, USA. Email: [jsiddiqui@umc.edu](mailto:jsiddiqui@umc.edu)

**Figure S1**


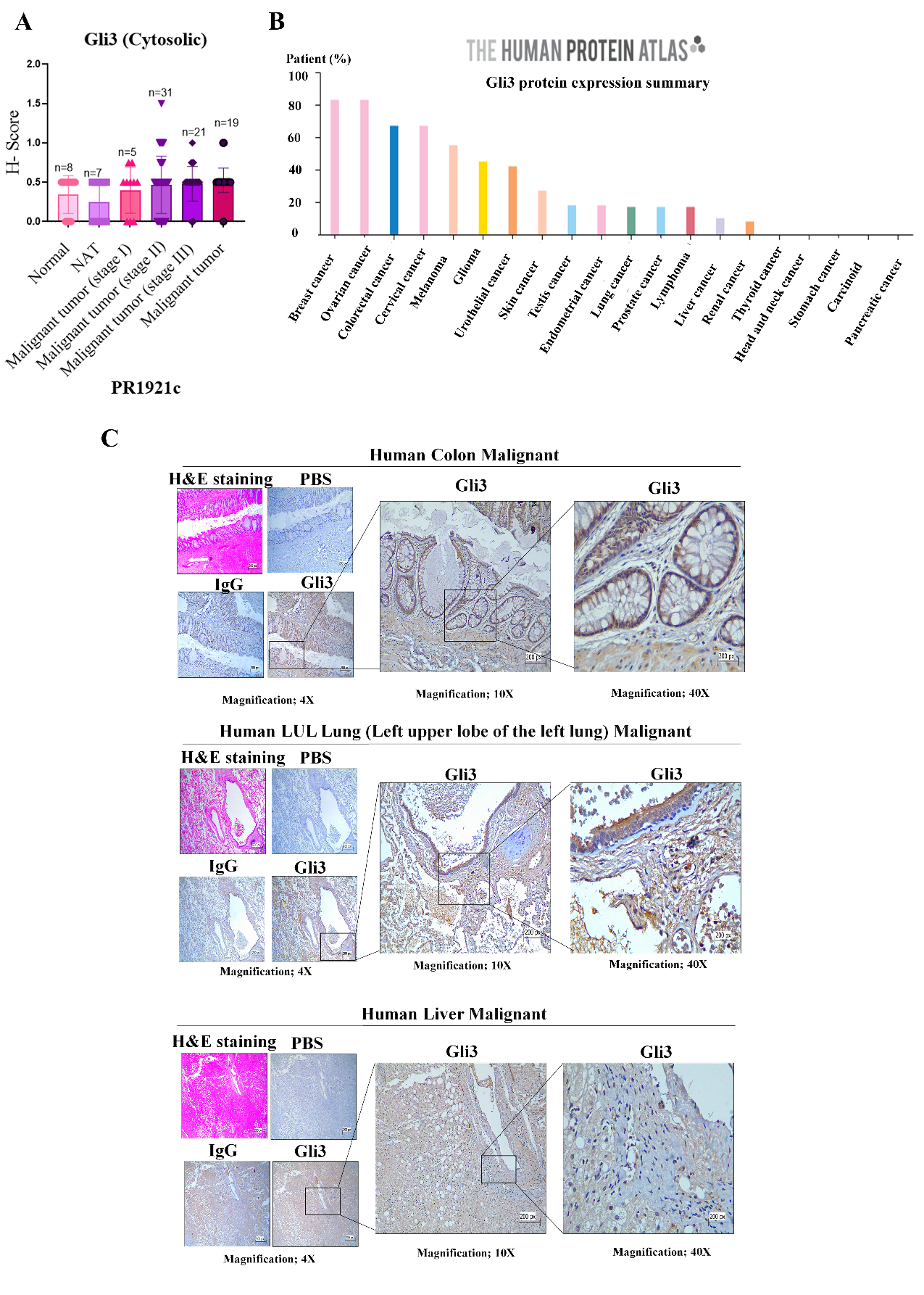


**Figure S1: Quantitative analysis of Gli1 in normal and malignant prostate tissues, along with validation of Gli3 in various malignant tissues (colon, lung, and liver) using isotype and control standards. (A)** Graphs correspond to the quantification results of cytosolic Gli3 protein expression in the indicated malignant tumor stages vs. normal, and n represents the number of patient samples in each group. Data are presented as mean ± SD. **(B)** Representative bar graph showing Gli3 protein expression across various human tissues, ranked from highest to lowest (Source: Human Protein Atlas; <https://www.proteinatlas.org> ). **(C)** Representative IHC images illustrating Gli3 expression, including negative control (PBS) and isotype control (IgG), across various selected tissues, as indicated in the figures.

**Figure S2**


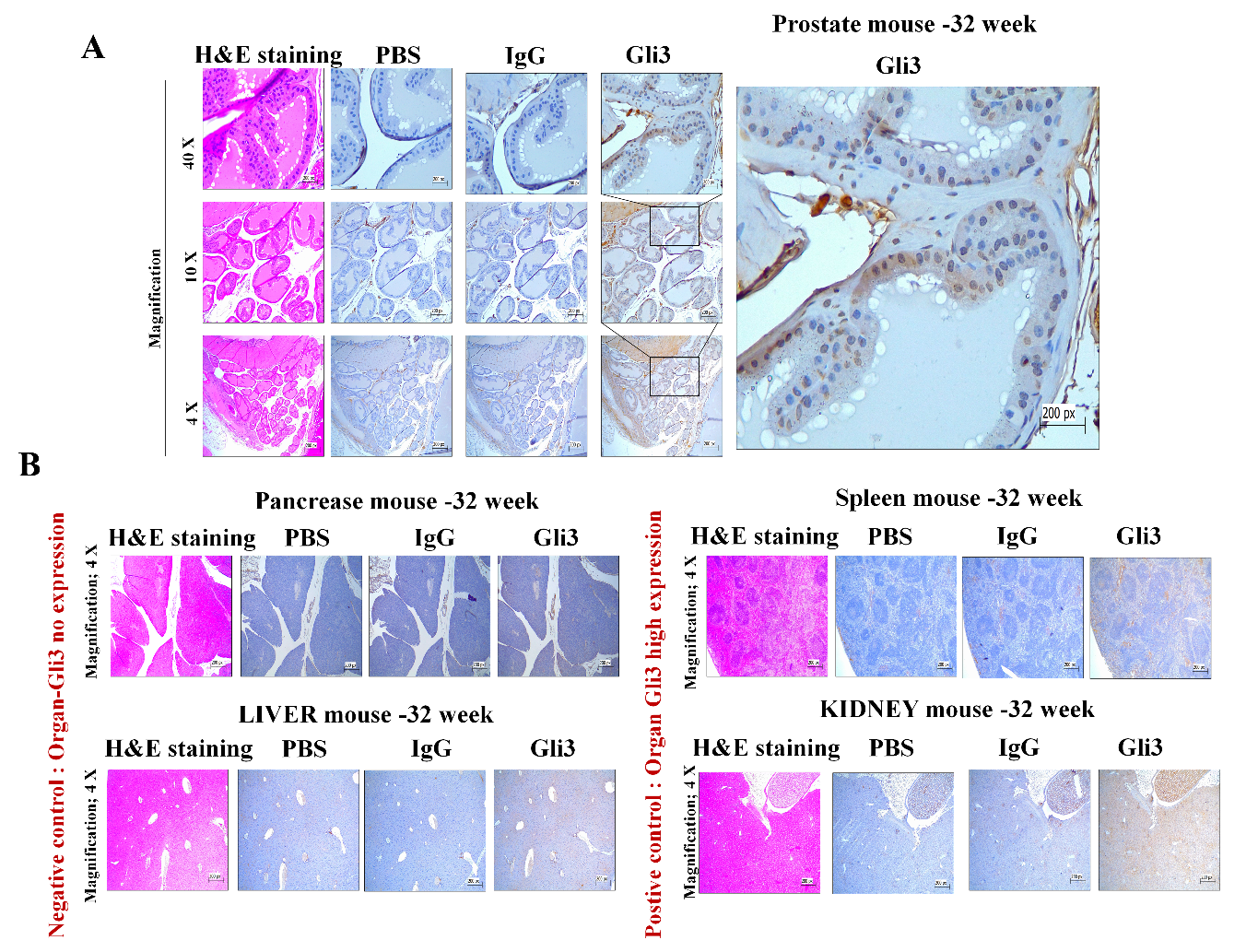


**Figure S2 Confirmation of Gli3 expression in mouse prostatic tissues using isotype controls and positive/negative control samples**. **(A, B)** H&E staining of representative mouse tissues alongside IHC demonstration of Gli3 protein in various normal mouse tissues, including prostate, pancreas, liver, spleen, and kidney.

**Figure S3**


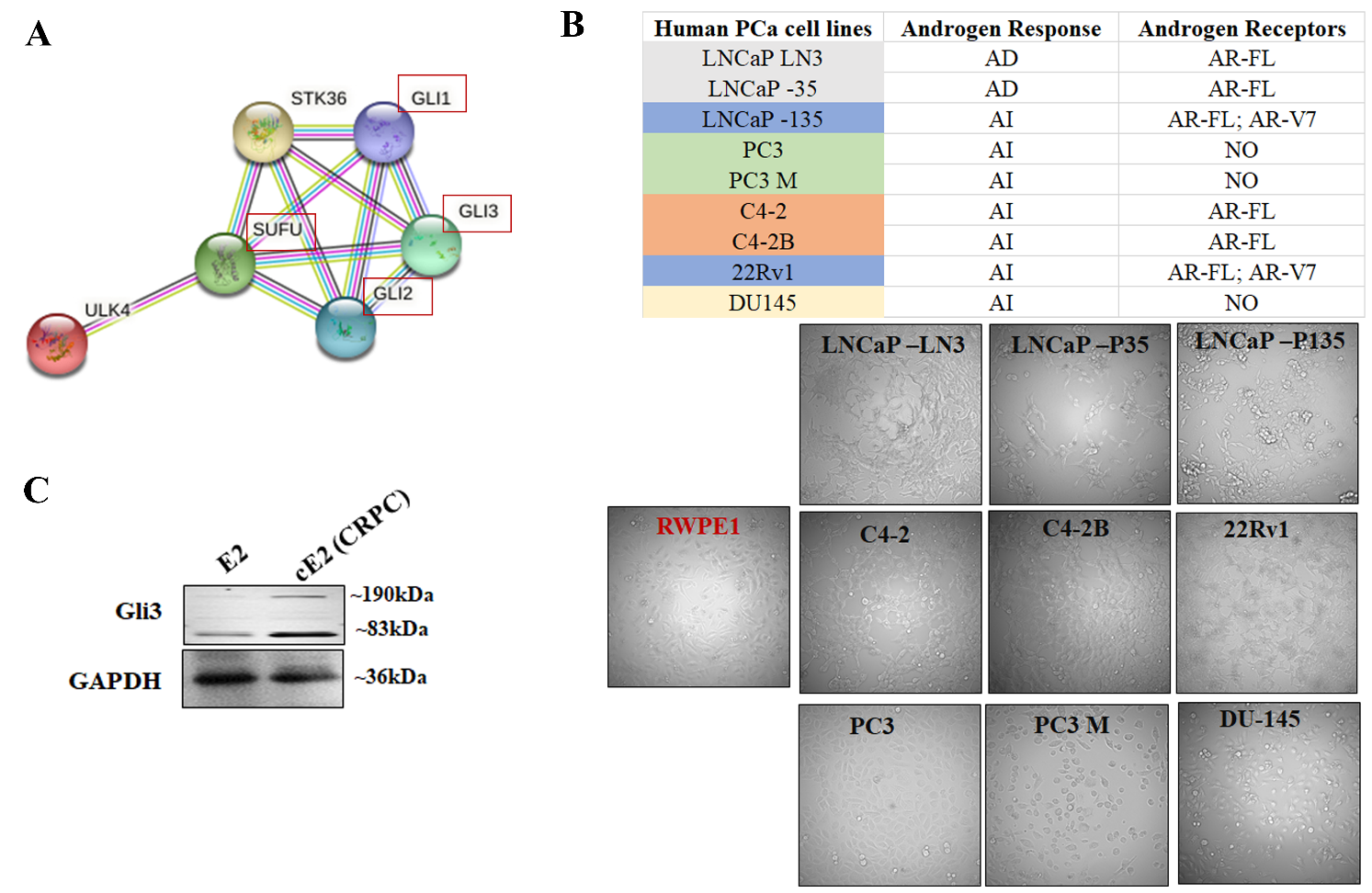


**Figure S3 STRING analysis of Gli family protein interaction, morphology of human prostatic (normal/ cancerous) cells with varying AR status, and Gli3 protein expression in mouse syngeneic PCa cell lines. (A)** STRING protein-protein interaction (PPI) network of Gli proteins with key regulatory proteins. **(B)** The table represents the androgen response and androgen receptor status in human PCa cell lines (upper panel). Representative images show the morphology of indicated cell lines (lower panel). **(C)** Representative WB images show the expression in syngeneic cell lines derived from non-castrated and castrated mice. GAPDH was used as a loading control.

**Figure S4**


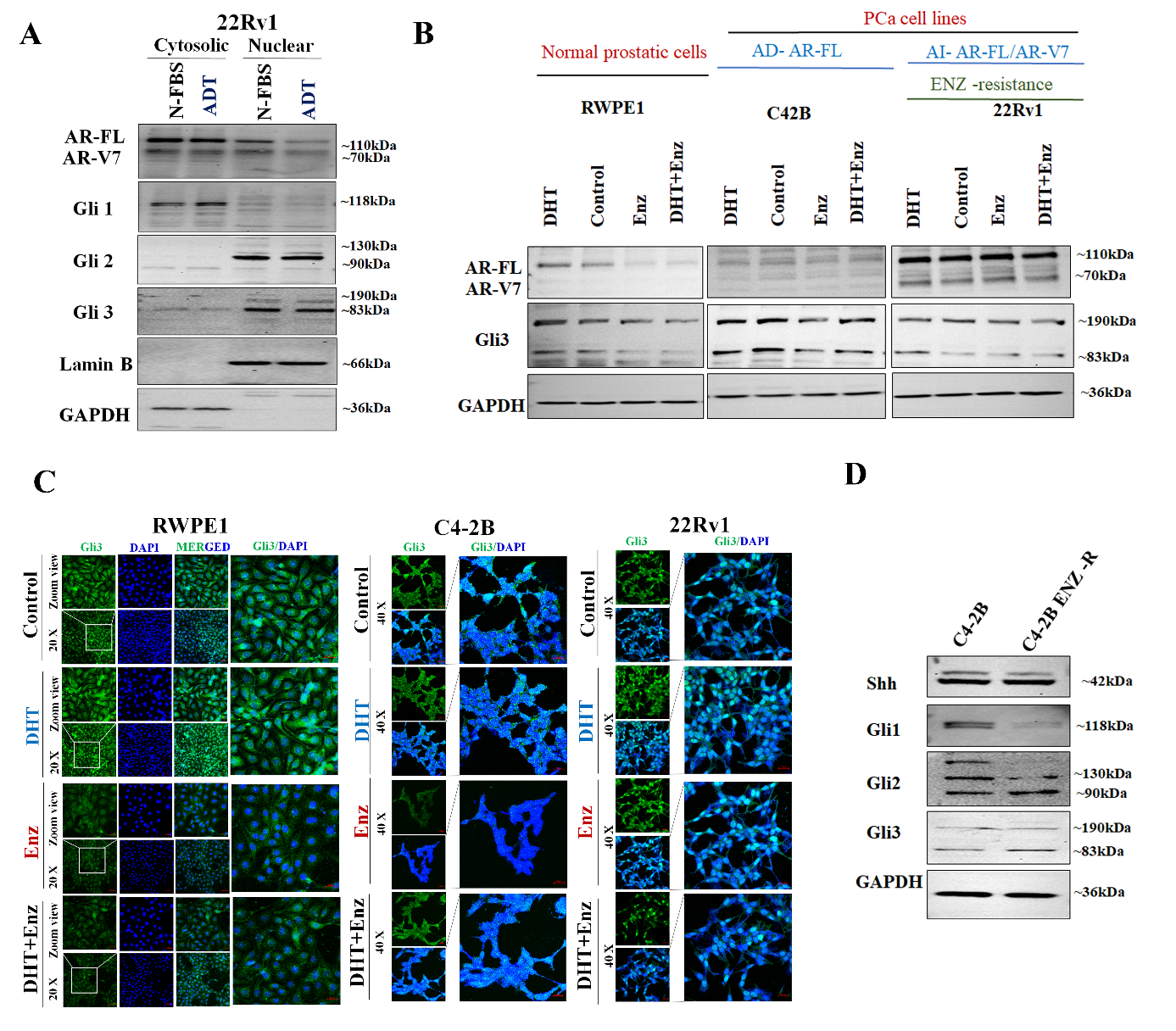


**Figure S4 Analysis of potential involvement of AR and Gli3 through androgen deprivation and treatment with AR agonist/antagonist: (A)** Representative WB images show the expression of AR (AR-FL and AR-V7) and Gli proteins ( Gli1, Gli2, and Gli3) in the cytosolic and nuclear fraction of 22Rv1 cells after being starved with charcoal-stripped FBS (ADT). GAPDH was used as a loading control. **(B)** Representative WB images show the expression of AR (AR-FL and AR-V7) and Gli3 proteins in normal (RWPE1) and PCa cell lines (C4-2B and 22Rv1) treated with the AR agonist (DHT - Dihydrotestosterone), the AR antagonist (ENZ - Enzalutamide), or a combination of both. GAPDH was used as a loading control. **(C)** Representative IF images displaying Gli3 expression in the indicated cells under various treatments. **(D)** Representative WB images showing the expression of Hh signaling proteins (Shh, Gli1, Gli2, and Gli3) in the C4-2B ENZ-resistant cell line compared to C4-2B. GAPDH was used as a loading control.

**Figure S5**


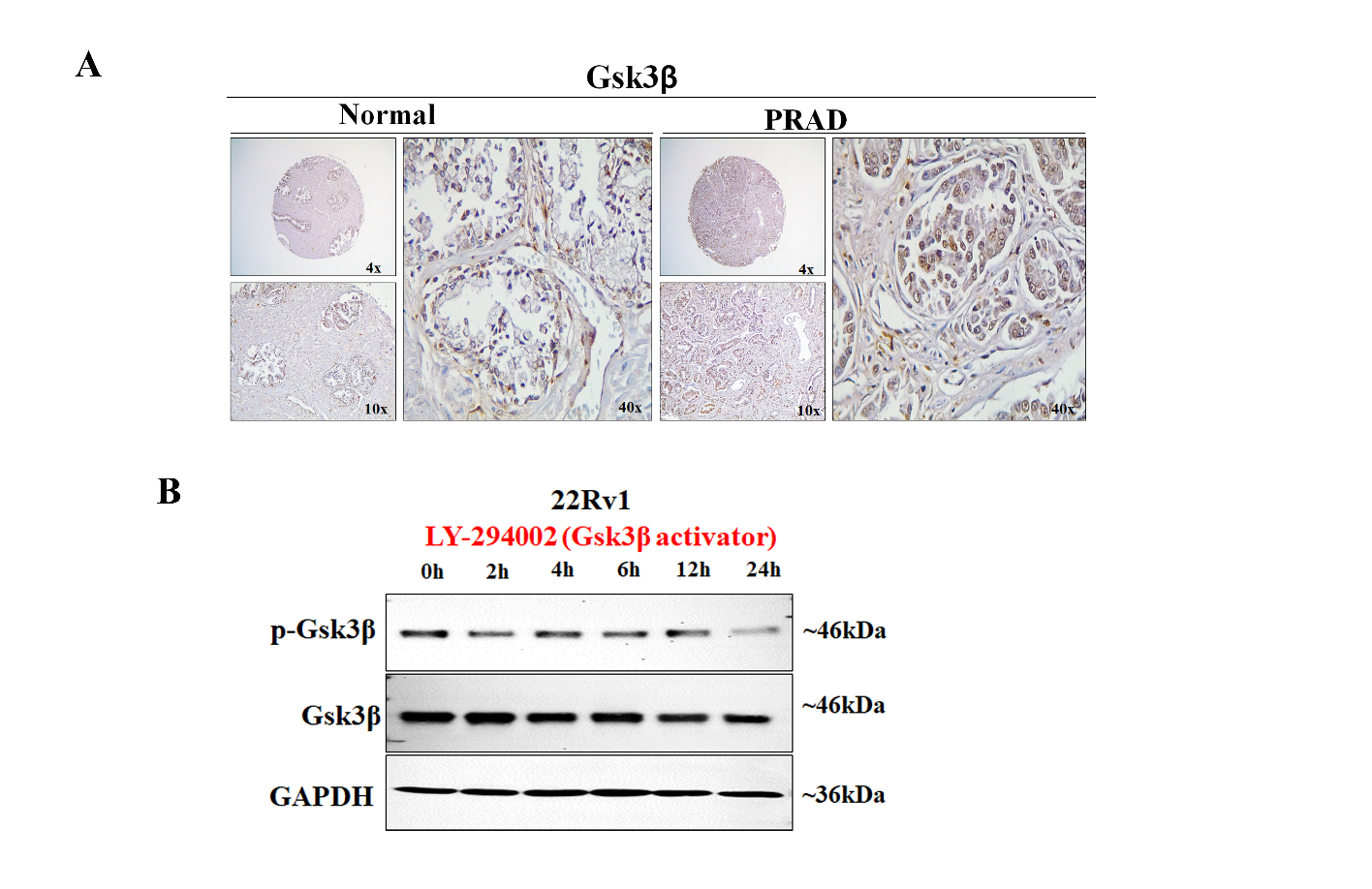


**Figure S5: Demonstration of the expression of Gsk3β in clinical samples and effect of Gsk3β activator in CRPC cells: (A)** Representative IHC images showing Gsk3β protein in human TMA containing samples from normal and malignant patients, as indicated. **(B)** Representative WB images show the expression of p-Gsk3β and Gsk3β in 22Rv1 cells treated with Gsk3β activator**.** GAPDH was used as a loading control.

**Figure S6**


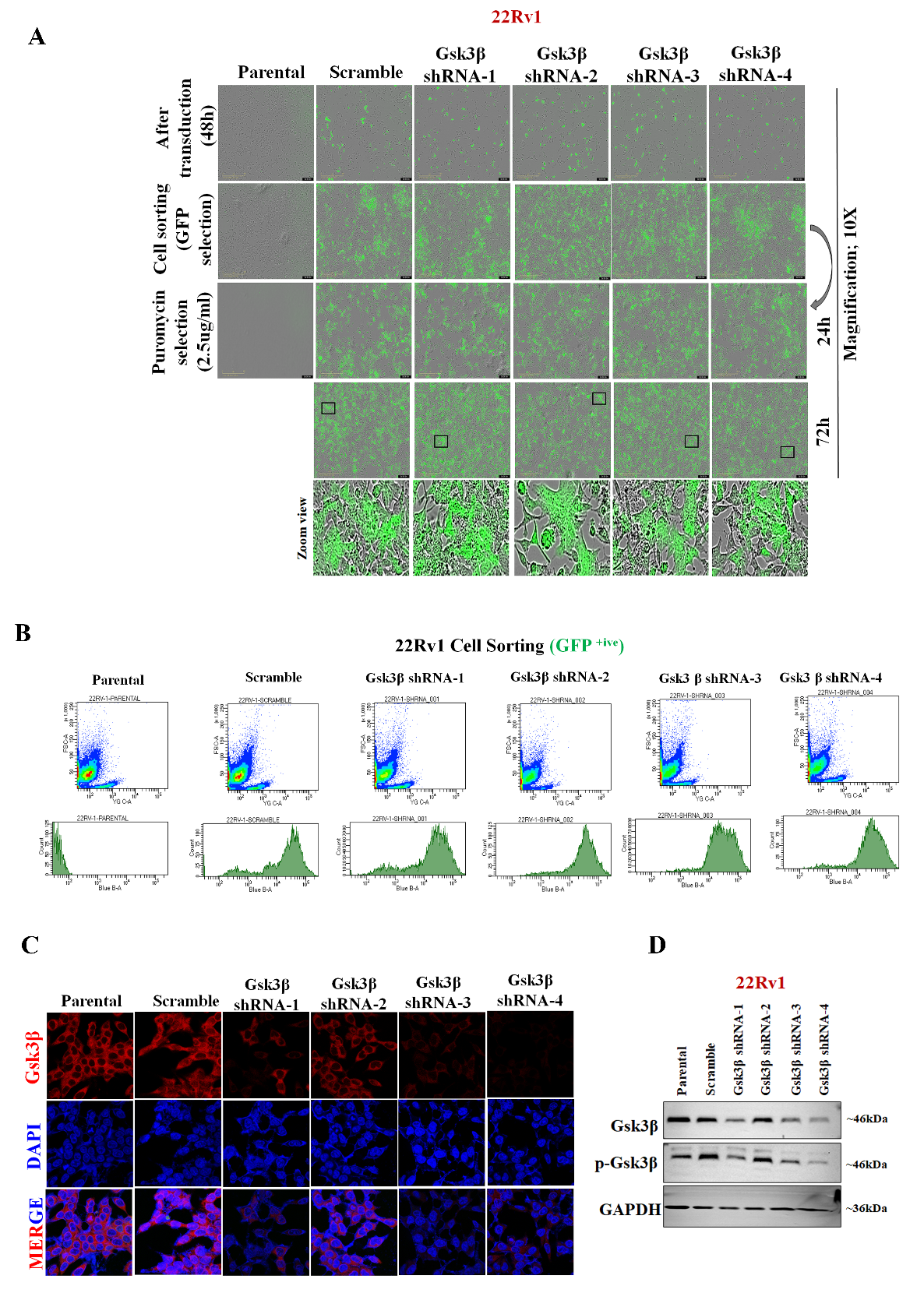


**Figure S6: Genetic blockage of Gsk3β in 22Rv1 using Gsk3β shRNA and corresponding non-targeting oligonucleotides and their effectiveness: (A)** Representative fluorescence images show the transduction efficiency of each clone, as indicated. **(B)** Representative micrographs show the flow cytometry cell sorting. **(C)** Representative immunofluorescence images show the expression of Gsk3β in 22Rv1 cells, as presented. **(D)** As indicated, representative WB images show the expression of p-Gsk3β and Gsk3β in 22Rv1 cells. GAPDH was used as a loading control.

**Figure S7**


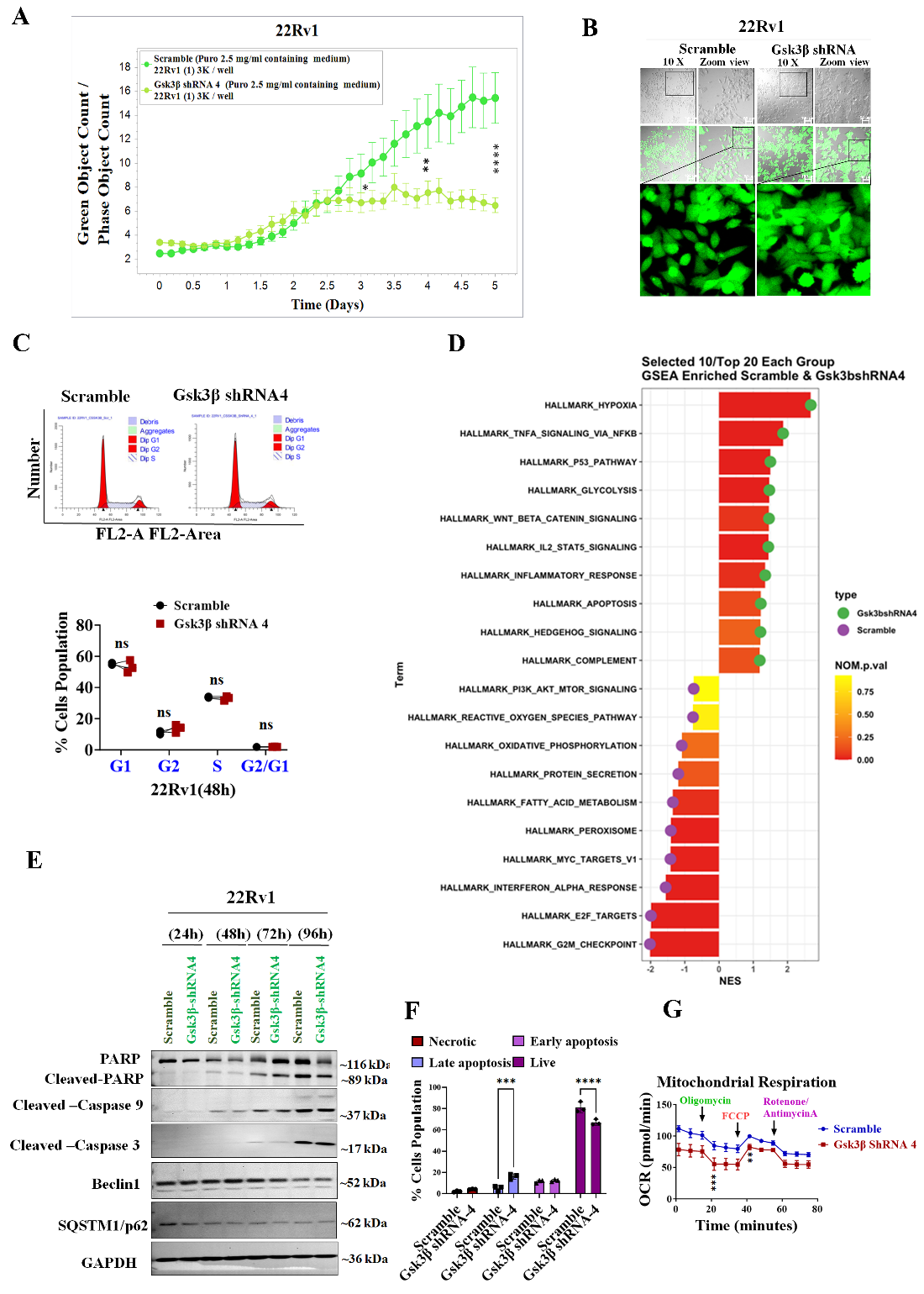


**Figure S7: Functional impact of genetic blockage of Gsk3β in 22Rv1 cells: (A)** Incucyte adherent cell-by-cell analysis demonstrates a time-dependent decrease in the proliferation of 22Rv1 Gsk3β KD compared to scramble cells. **(B)** Representative fluorescence images show the morphology of 22Rv1 scramble and Gsk3β KD cells. **(C)** 22Rv1 Gsk3β KD and scramble cells stained with propidium iodide (PI) followed by flow cytometry to determine the cell cycle distribution based on DNA content (upper panel). Quantitative analysis of these micrographs was shown as mean ± SEM (lower panel). **(D)** The GSEA analysis revealed ten major upregulated and downregulated pathways through GSEA analysis, selected from the top 20 pathways associated with metabolism and growth. **(E)** Representative WB images show the expression of apoptotic and autophagic proteins in 22Rv1 Gsk3β KD and scramble cells in a time-dependent manner. GAPDH was used as a loading control. **(F)** Quantitative apoptosis analysis of FACS was shown as mean ± SEM (n = 3). **(G)** OCR analysis of Gsk3β KD vs. scramble cells.

**Figure S8**


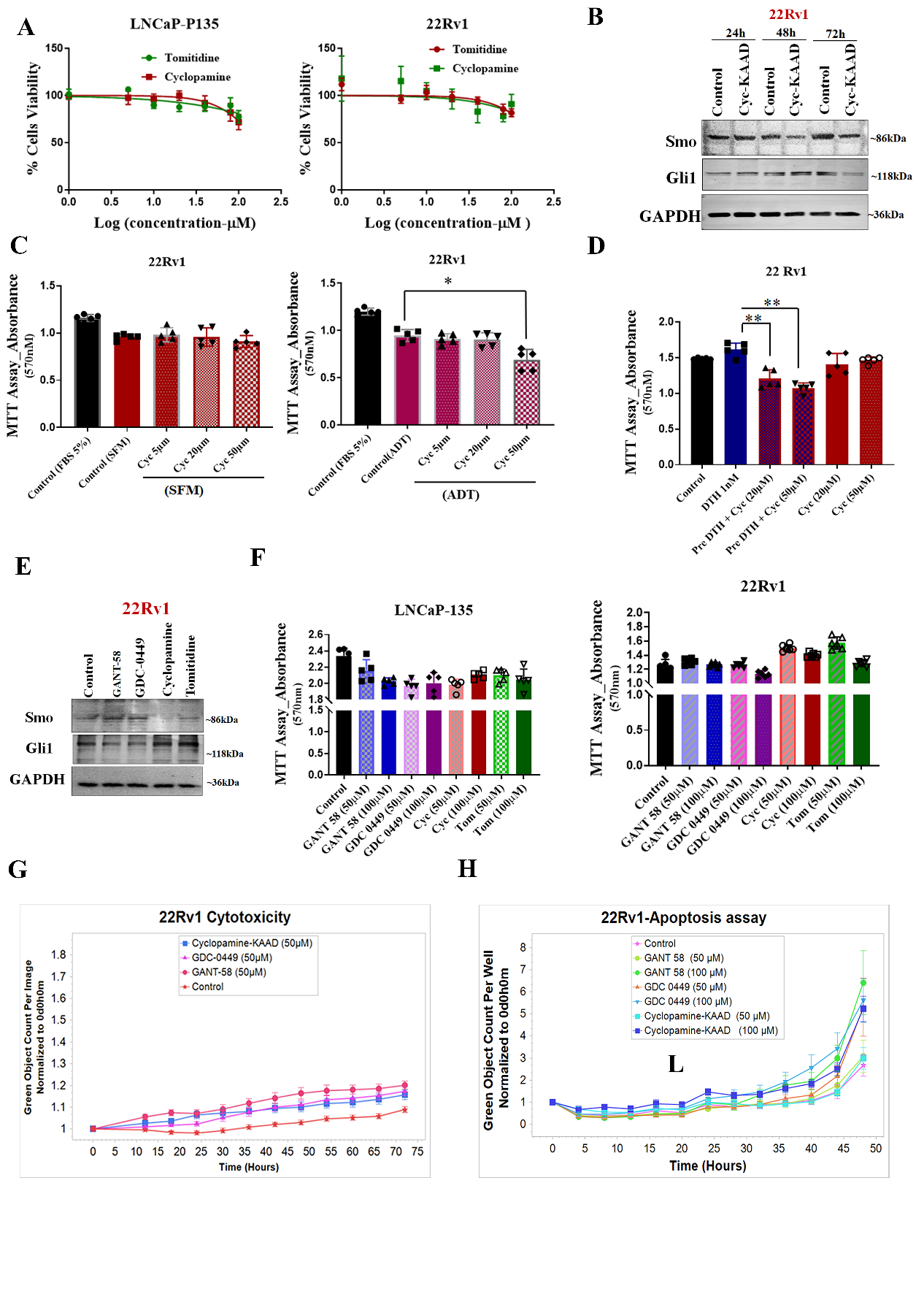


**Figure S8: Effect of inhibitors of Smo/Gli1 cascade on CRPC cell viability: (A)** Concentration-dependent effect of Smo inhibitor, Cyclopamine, and its analog Tomitidine treatment on the viability of CRPC cells (LNCaP-135 and 22Rv1). **(B)** Representative WB images show the expression of Smo and Gli1 in 22Rv1 cells treated with Cyclopamine in a time-dependent manner. GAPDH was used as a loading control. (**C)** The concentration-dependent effect of Cyclopamine treatment on the viability of 22Rv1 during SFM and ADT conditions using MTT assay. Values are expressed as mean ± SEM (n = 5), p values: * p < 0.05 vs. control cells. **(D)** The viability of 22Rv1 cells treated with Cyclopamine, pre-treated with DTH and combination, is assessed in the indicated groups via MTT assay after 48 h incubation. **(E)** Representative WB images show the expression of Smo and Gli1 in 22Rv1 cells treated with different Smo/Gli1 cascade inhibitors (Cyclopamine, GDC-0449, and GANT-58). GAPDH was used as a loading control. Values are expressed as mean ± SEM (n = 5), p values: ** p < 0.01 vs. DTH-treated cells. **(F)** The effect of inhibitors cyclopamine, GDC-0449, and GANT-58 treatment on the viability of CRPC cells (LNCaP-135 and 22Rv1) as presented concentrations. **(G, H)** Incucyte adherent cell-by-cell analysis demonstrates time-dependent viability (left panel) and apoptosis (right panel) of 22Rv1 cells treated with Cyclopamine, GDC-0449, and GANT-58 as indicated concentrations.

**Figure S9:**


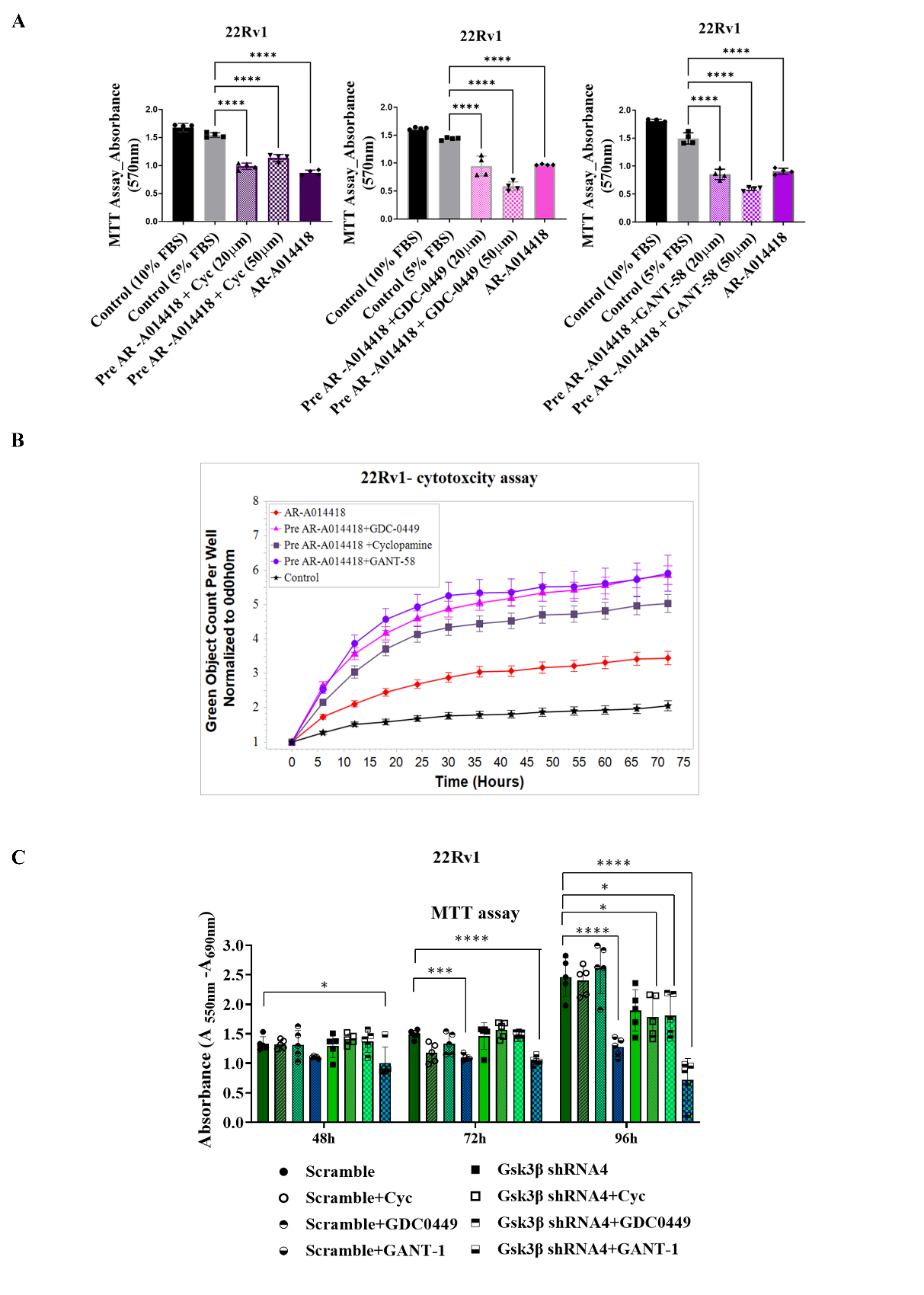


**Figure S9: Impact of combinational treatment on CRPC cell viability: (A)** The graph illustrates the cell viability of 22Rv1 cells pre-treated with Gsk3β inhibitor (AR-A014418) along with selective Smo/Gli1 cascade inhibitors (Cyclopamine, GDC-0449, and GANT-58) using the MTT assay after a 48-hour incubation. **(B)** Incucyte adherent cell-by-cell analysis demonstrates a time-dependent decrease in the proliferation of 22Rv1 cells treated with inhibitors, as indicated. **(C)** The graph represents the cell viability of 22Rv1 Gsk3β KD or scramble cells treated with selective Smo/Gli1 cascade inhibitors (Cyclopamine, GDC-0449, and GANT-58) using the MTT assay after 48 h incubation.
