# Supplementary material for: Oncogenic potential of truncated-Gli3 via the Gsk3β/Gli3/AR-V7 axis in castration-resistant prostate cancer
Source: Oncogene. 2025 Jan 16;44(15):1007–23. doi: 10.1038/s41388-024-03266-z (PMC11976299; doi:10.1038/s41388-024-03266-z)
Supplement: Supplementary file 2 — Supplementary Table [file 41388_2024_3266_MOESM2_ESM.docx]

**Oncogenic Potential of Truncated-Gli3 via the Gsk3β/Gli3/AR-V7 Axis in Castration-Resistant Prostate Cancer**

**Supplementary table:** List of antibodies, chemicals, biochemical assay kits, reagents, cell lines, and other relevant details utilized in this study.

| **REAGENT or RESOURCE** | **SOURCE** | **IDENTIFIER** |
| --- | --- | --- |
| **Antibodies** | | |
| Anti-Gli1 antibody | Santa Cruz Biotechnology | Cat# sc-515781 |
| Anti-Gli2 antibody | Santa Cruz Biotechnology | Cat# sc-271786 |
| Anti-Gli3 antibody | Thermo Fisher Scientific | Cat# 19949-1-AP |
| Anti-Gli3 antibody | Santa Cruz Biotechnology | Cat# sc-74478 |
| Anti-Gli3 antibody | Rockland/Thermo Fisher Scientific | Cat# 600-401-694 |
| Anti-Gli3 antibody | Invitrogen/ Thermo Fisher Scientific | Cat# PA5-28029 |
| Anti-Sufu antibody | Invitrogen/ Thermo Fisher Scientific | Cat# PA5-29952 |
| Anti-Shh antibody | Santa Cruz Biotechnology | Cat# sc-373779 |
| Anti-Smo antibody | Invitrogen/ Thermo Fisher Scientific | Cat# PA5-113312 |
| Anti-Ptch antibody | Invitrogen/ Thermo Fisher Scientific | Cat# PA5-87508 |
| Anti-Ace-α-tubulin antibody | Cell Signaling Technology | Cat# 5335T |
| Anti-Ace-α-tubulin antibody | Cell Signaling Technology | Cat# 12152s |
| Anti- α-tubulin antibody | Cell Signaling Technology | Cat# 2144s |
| Anti- Gsk3β antibody | Cell Signaling Technology | Cat# 12456s |
| Anti- p-Gsk3β antibody | Cell Signaling Technology | Cat# 9322s |
| Anti-AR antibody | Invitrogen/ Thermo Fisher Scientific | Cat# MA5-13426 |
| Anti-p-Akt | Cell Signaling Technology | Cat# 4060s |
| Anti-Akt antibody | Cell Signaling Technology | Cat# 4691s |
| Anti-Bcl2 antibody | Invitrogen/ Thermo Fisher Scientific | Cat# PA5-27094 |
| Anti-Bax antibody | Cell Signaling Technology | Cat# 89477s |
| Anti-cleaved caspase-3 antibody | Cell Signaling Technology | Cat# 9661s |
| Anti-cleaved caspase-9 antibody | Cell Signaling Technology | Cat# 20750s |
| Anti-PARP antibody | Cell Signaling Technology | Cat# 9542s |
| Anti-Beclin-1 antibody | Santa Cruz Biotechnology | Cat# sc-11427 |
| Anti-P62 antibody | Cell Signaling Technology | Cat# 5114s |
| Anti-Lamin B1 antibody | Cell Signaling Technology | Cat# 12586s |
| Anti-Histone H3 antibody | Abcam | Cat# ab1791 |
| Anti-GAPDH antibody | Cell Signaling Technology | Cat# 5174s q |
| Universal HRP-conjugated Secondary antibody | Vector Laboratories | Cat# MP-7500 |
| HRP-conjugated Rabbit secondary antibody | Invitrogen | Cat# 32460 |
| HRP-conjugated Mouse secondary antibody | Invitrogen | Cat# 31430 |
| Goat anti-Rabbit IgG (H+L) Secondary Antibody, Alexa Fluor 568 | Invitrogen | Cat# A-11011 |
| Goat anti-Mouse IgG (H+L) Secondary Antibody, Alexa Fluor 568 | Invitrogen | Cat# A-11004 |
| Goat anti-Rabbit IgG (H+L) Secondary Antibody, Alexa Fluor 488 | Invitrogen | Cat# A-11034 |
| Goat anti-Mouse IgG (H+L) Secondary Antibody, Alexa Fluor 488 | Invitrogen | Cat# A-11001 |
| Goat anti-Rabbit IgG (H+L) Secondary Antibody, Alexa Fluor 647 | Invitrogen | Cat# A-21245 |
| Goat anti-Mouse IgG (H+L) Secondary Antibody, Alexa Fluor 647 | Invitrogen | Cat# A-21235 |
| **Chemicals and Inhibitors** | | |
| Cyclopamine-KAAD | Millipore Sigma | Cat# 239804-100UG; CAS# 306387-90-6 |
| Tomatidine | EMD-Milipore | Cat# 614350-25MG; CAS# 6192-62-7 |
| GDC-0449 | APExBIO | Cat# A3021-50MG |
| GANT-58 | Sigma-Aldrich | Cat# 64048-12-0-5MG; CAS # 64048-12-0 |
| LY-294002 | EMD-Milipore | Cat# 440202; CAS# 154447-36-6 |
| AR-A014418 | Sigma-Aldrich | Cat# A3230-5MG; CAS#  487021-52-3 |
| Enzalutamide | Med Chem Express (MCE) | Cat# HY 70002/CS-0317 |
| Dihydrotestosterone | Sigma-Aldrich | Cat# 730637-1ML |
| Protein A/G plus agarose bead | Santa Cruz Biotechnology | Cat# sc-2003 |
| Lipofectamine 2000 | ThermoFisher | Cat# 11668027 |
| DMSO | Sigma | Cat# D2650 |
| Matrigel | Corning | Cat# 356234, 356,255 |
| LightCycler 480 SYBER Green I Master mix | Roche | Cat# 04707516001 |
| Propidium Iodide | ThermoFisher | Cat# P1304MP |
| IncuCyte Annexin V Green dye | Sartorius | Cat# 4624 |
| IncuCyte cytotox green reagent | Sartorius | Cat# 4633 |
| Pierce™ ECL Western Blotting Substrate | ThermoFisher | Cat# 32106 |
| DAPI Fluoromount-G | SouthernBiotech | Cat# 0100-20 |
| Seahorse XF Cell  Mito Stress Test Kit | Agilent | Cat# 103015-100 |
| Seahorse XF RAPI media | Agilent | Cat# 103576-100 |
| Seahorse XF 1.0 M Glucose Solution | Agilent | Cat# 103577-100 |
| Seahorse XF 100mM Pyruvate Solution | Agilent | Cat# 103578-100 |
| Seahorse XF 200mM Glutamine Solution | Agilent | Cat# 103579-100 |
| MitoTracker Deep Red FM | ThermoFisher | Cat# M22426 |
| **Critical commercial assays** | | |
| iScript cDNA Synthesis Kit | Bio-Rad | Cat# 1708891 |
| ChIP -Kit | Abcam | Cat# ab500 |
| **Deposited data** | | |
| RNA-sequencing data | Gene Expression Omnibus (GEO) | Accession no. GSE255648 |
| **Experimental models: Cell lines** | | |
| RWPE1 | ATCC | Cat# CRL-3607 |
| 22Rv1 | ATCC | Cat# CRL-2505 |
| LNCaP-33 | Gifted | ---- |
| LNCaP-135 | Gifted | ---- |
| LNCaP-LN3 | Gifted | ---- |
| PC3 | ATCC | Cat# CRL-1435 |
| PC3M | Gifted | ---- |
| DU-145 | ATCC | Cat# HTB-81 |
| C4-2 | ATCC | Cat# CRL-3314 |
| C4-2B | ATCC | Cat# CRL-3315 |
| **Experimental models: Organisms/strains** | | |
| Athymic Nude Mouse | Envigo | N/A |
| Human Prostate Cancer Tissue Microarray | US Biomax | Cat# T191a; Cat# PR1921c |
| MYC^OE^ mouse prostate tissue | Gifted | Dr. Parthasarathy |
| **Oligonucleotides** | | |
| Primers for ChIP | Eurofins | NKX3-1(ARE-1)  5′-TTGCATAAATTAGGGGAGAACATACCA-3′;  5′-GAGGGACCCAGCTGCGATTCA-3′  PSA (ARE)  5′-CCTAGATGAAGTCTCCATGAGCTACA-3;  5′-GGGAGGGAGAGCTAGCACTTG-3′ |
| **Recombinant DNA** | | |
| ON-TARGETplus Human GLI3 siRNA and corresponding non-targeting controls (Scr) | Dharmacon/horizon | Cat# L-011043-00-0005 |
| Human Gsk3β-shRNA lentiviral particles (4 target specific, and 1scramble control | Origene | Cat# TL320376V |
| **Software and algorithms** | | |
| ImageJ | ImageJ | <https://imagej.net/software/imagej/> |
| GraphPad Prism 10 | GraphPad | <https://www.graphpad.com/> |
