# Supplementary material for: Oncogenic potential of truncated-Gli3 via the Gsk3β/Gli3/AR-V7 axis in castration-resistant prostate cancer
Source: Oncogene. 2025 Jan 16;44(15):1007–23. doi: 10.1038/s41388-024-03266-z (PMC11976299; doi:10.1038/s41388-024-03266-z)
Supplement: Supplementary file 3 — Supplementary Material and Methods [file 41388_2024_3266_MOESM3_ESM.docx]

**­SUPPLEMENTARY MATERIALS AND METHODS**

**Oncogenic Potential of Truncated-Gli3 via the Gsk3β/Gli3/AR-V7 Axis in Castration-Resistant Prostate Cancer**

1. **MTT Assay:**

2.5 × 10^3^ cells/well were seeded in 96-well plates 16-24h before the treatment. The cells were incubated with increasing concentrations of different treatments for various time points. Following treatment, cells were incubated with MTT (0.5 mg/mL) for 3 hrs. The medium was discarded, and 200 μL/well DMSO was added. The optical density (OD) was determined by measuring the absorbance at 570 nm using a plate reader (BMG Labtek) [1].

1. **Transfection: Stable and Transient Knockdown**

The 22RV1 cells with stable Gsk3β knockdown (KD) and scramble control were generated using a human shRNA (Gsk3β shRNA1, 2, 3, and 4) lentiviral particle system from ORIGENE. The lentiviral and polybrene mixture were used in each well for transduction. 72 hrs after transfection, GFP+-transfected 22Rv1 cells were sorted using FACS Aria II (BD Biosciences) (Figure 9 A, B). Sorted GFP-positive cells were subsequently selected for puromycin-containing media (2.5 μg/mL). We also checked the knockdown efficiency via WB and noticed that shRNA1 and 4 were more effective.

The transient siRNA-mediated Gli3 KD was performed in 22Rv1 cells by transfecting with either target siRNA (SMARTPool, concentration 50 nmol·L−1) or control siRNA using ON-TARGET PLUS siRNA reagents (Dharmacon, Illinois, USA) by following manufacturer’s instructions [2]. The KD efficiency was tested using a western blot.

1. **Confocal Microscopy**

Cells were seeded on coverslips in a 12-well plate for immunostaining, fixed using ice-cold methanol, and permeabilized with the 0.1% Triton X-100. Cells were washed with PBS, blocked with 2% BSA, and incubated with primary antibodies overnight at 4 °C. Following primary antibody incubation, the cells were incubated at room temperature for 1 hour with fluorescence-tagged secondary antibodies (FITC, Cy-3), then mounted on slides with VECTASHIELD-containing 4′,6-diamidino-2-phenylindole (DAPI) (Vector Laboratories, Burlingame, CA, USA. Confocal images were collected using a Zeiss LSM 800/ LSM 710 confocal microscope with a 63×/1.4 NA or 40× NA oil objective [3][4].

1. **Quantification of Fluorescent Intensity**

Image J analysis was used to quantify the intensity of interested protein expression in the human PCa cell lines, as well as in patient and mouse tissue samples. Details of each experiment are explained in the respective figure legends. In brief, multichannel snapshots were split into separate channels using ImageJ. A region of interest was delineated around individual cells in one of the two or three channels using the "freehand" tool. This region was then analyzed using measure plugins such as colocalization finder and JACoP, with intensity calculated by multiplying area and mean intensity. Intensity measurements were conducted for five cells in a field and five images per condition. The intensity values were represented relative to the control condition in arbitrary units [5].

1. **Cilia Staining:**

The cells were seeded and fixed using 4% paraformaldehyde to perform cilia staining with localization of interested proteins. Subsequently, they were blocked in PBS containing 3% BSA. Next, the cells were subjected to overnight incubation with the primary anti-acetylated α-tubulin antibody, along with Smo, Ptch, or Gli3 antibodies. Following the primary antibody incubation, fluorescence-tagged secondary antibodies were applied for 1 hour. The cells were then mounted on slides using VECTASHIELD containing 4′,6-diamidino-2-phenylindole (DAPI) to stain the nuclei. Finally, the stained cells were imaged using a Zeiss LSM 710 (20×) confocal microscope [6].

1. **Mitochondrial Imaging**

Cells were seeded on coverslips in a 12-well plate and then stained with 100 nM of MitoTracker Red for 30 min at 37 °C in the dark as per published protocol [3]. Following staining, cells were washed with PBS, fixed with 4% paraformaldehyde, and counterstained with VECTASHIELD-containing DAPI, and images were captured at 40× NA oil or 20× objective using Carl Zeiss microscope (LSM 710 META) [1].

1. **Immunoprecipitation**

Protein samples were incubated with protein A/G plus agarose beads for 1–2 h to pre-clarify, followed by washing and centrifugation. Pre-cleared lysates were incubated with specific antibodies and isotype control along with 30 μL of protein A/G plus agarose beads overnight at 4 °C with continuous agitation. The next day, incubated samples were centrifuged, and pellets were washed and resuspended with RIPA lysis buffer. The immunoprecipitated protein sample, input (3–5% of the total protein), with input (3–5% of the total protein), and IgG control samples prepared in Laemmli buffer were separated using SDS-PAGE [1].

1. **Nuclear and Cytoplasmic Extraction**

To assess the localization of Hh/Gli signaling components in prostate cancer (PCa) cells, nuclear and cytoplasmic fractions were isolated using extraction buffers with a protease inhibitor cocktail, as published protocol [1]. Cells were scraped, washed in 1× PBS, and lysed for the cytoplasmic fraction with a harvest buffer (10 mM HEPES pH 7.9, 50 mM NaCl, 0.5 M sucrose, 0.1 M EDTA and 0.5% triton X 100). Cytoplasmic proteins were separated by centrifugation, and nuclei pellets were washed with Buffer A before lysis (10 mM N-(2-Hydroxyethyl)piperazine-N′-(2-ethane sulfonic acid) (HEPES) pH 7.9, 10 mM potassium chloride (KCl), 0.1 mM ethylenediaminetetraacetic acid (EDTA), and 0.1 mM ethylene glycol-bis (β-aminoethyl ether)-N, N, N′, N′-tetraacetic acid (EGTA)) and lysed by using Buffer C (10 mM HEPES pH 7.9, 50 mM sodium chloride (NaCl), 0.1 mM EDTA, 0.1 mM EGTA and 0.1% nonidetP-40) following vigorous shaking for 15 min at 4 °C. Finally, both cytoplasmic and nuclear fractions were centrifuged (14,000 rpm for 15 min at 4 °C) and subjected to Western blot analysis [1].

1. **Chromatin immunoprecipitation (ChIP) assay**

Cells were washed, harvested, and processed using a ChIP assay kit per manufacturers' instructions with minor modifications followed by RT-PCR [7]. For input control, 10% of the sonicated samples had been separated. Immunoprecipitation was performed with ChIP-grade mouse monoclonal anti-AR antibody. Samples were washed and eluted as per instructions. The purified DNA was then subjected to PCR amplification.

1. **Real-time growth kinetics on PCa cells**

Cells were seeded in 96-well plates and treated with respective drugs as indicated, followed by staining the cells with IncuCyte cytotox green reagent for counting dead cells (Sartorius, 4633) and IncuCyte Annexin V Green dye (Sartorius, 4624) for apoptosis. Fluorescent objects were quantified in real-time to identify cell death or apoptosis via IncuCyte integrated analysis software (Sartorius). Fluorescent and phase images taken on the same vessels were to normalize and quantify cell proliferation/ growth using the IncuCyte cell-by-cell analysis software module (Sartorius). Real-time images of the cells were captured every 6 hrs for 5-9 days and were analyzed and graphically presented using the Incucyte Live-Cell Imaging analysis system (Essen Bioscience) [8].

Notably, no external dyes were used for GFP-sorted 22Rv1- scramble and Gsk3β KD cell proliferation and processed as earlier described method. All the experiments were graphically

presented using IncuCyte software (Sartorius).

1. **Immunohistochemistry and Immunofluorescence**

Tissue microarrays (TMAs) (Cat# PR1921c 96 cases/192 cores; T191a 6 cases/24 cores), including tissue samples from normal prostate and malignant tumors, were purchased from US Biomax. TMAs were subjected to immunohistochemical staining with the primary antibodies, as provided in the supplementary table. Antigen retrieval was performed in 10 mM sodium citrate buffer (pH 6) and followed the publication’s protocol [9][10]. The stained sections were scored by Dr. Subodh M. Lele (pathologist, UNMC) in a blinded fashion. The intensity of protein expression was graded on a scale of 0 to 3 (0, no staining; 1+, weakly positive; 2+, moderately positive; 3+, strongly positive). The percentage of positive staining was scored in the range of (0–100% or 0–1). A histoscore was calculated by multiplying intensity (0–3) and positivity (0–1), ranging between 0 and 3.

Immunofluorescence analysis of tissue sections was conducted using the method previously described [5].

1. **Western Blot**

Protein samples were harvested using RIPA lysis buffer (50 mM Tris-HCl, pH 7.4; 1% NP-40; 1% sodium deoxycholate; 0.1% sodium dodecyl sulfate (SDS), 150 mM NaCl; 2 mM EDTA; 25 mM sodium fluoride, sodium orthovanadate, and 1 mM phenylmethylsulfonyl fluoride (PMSF)) supplemented with 1× protease inhibitor cocktail (Roche). The protein lysates were quantified by the bicinchoninic acid (BCA) method and then prepared using laemmli buffer. 20–30 µg of protein was loaded and separated by 10% SDS-PAGE gel. Proteins were transferred to polyvinylidene difluoride (PVDF) membranes and then blocked in 5% non-fat dry milk in PBS containing 0.1% Tween 20 (PBST). Blots were incubated overnight at 4°C with primary antibodies, as detailed in the supplementary table. The membranes were then washed in PBST, probed with the appropriate secondary antibodies for 1 hour at room temperature, and then washed with PBST. GAPDH was used as a loading control for protein normalization. Signals were detected with the ECL chemiluminescence kit using iBright (Thermo Scientific) [1][8].

1. **Colony Formation (clonogenic assay)**

Cells were seeded at a density of 1000 cells/well in 6-well plates in triplicates. Cells were incubated for two weeks at 37 °C and 5% CO_2_, with media change every three days. After 14 days, cell colonies were fixed with ice-cold methanol and stained with crystal violet (0.125 gm of crystal violet in 50 mL of 20% methanol). Colonies were then dissolved in 10% glacial acetic acid for quantification, and the optical density was measured at 590 nm under a microplate reader [11].

1. **Apoptosis Assay**

22RV1- GSK3β scramble and KD **c**ells were cultured. Cell supernatant and adherent cells were collected, washed with PBS, and resuspended with calcium-binding buffer. Cells were then stained with annexin V and propidium iodide for 20 minutes at 37 °C and analyzed by FACS Canto™ flow cytometry [1].

1. **Cell-cycle analysis**

Cell cycle analysis was performed using propidium iodide (Roche Diagnostics) staining. Briefly, 22Rv1 Gsk3β KD and scramble cells were seeded (1 × 10^6^ cells), synchronized by double thymidine block, harvested, washed, and fixed in 70% ethanol. After fixation, the cells were left on ice for approximately 45 min, followed by centrifugation. The pellets were resuspended in Telford’s reagent (50 μg/mL propidium iodide, 90 mM EDTA, 0.1% Triton X-100, and 1μg/mL RNase A). The DNA content of stained cells was analyzed by using a FACS Canto™ flow cytometer [1].

1. **Seahorse Cell Mito Stress assay**

To assess the mitochondrial respiration rate of 22Rv1-Gsk3β shRNA4 and scramble cells, we employed the Agilent Seahorse XF Cell Mito Stress Test Kit (Cat# 103015-100) following the manufacturer's instructions [12]. Cells were seeded at a density of 3 × 10^4^ based on their optimal response to the kit reagents. The analysis of mitochondrial function in this assay encompasses various parameters, including basal respiration, ATP-linked respiration, maximal and reserve capacities, and non-mitochondrial respiration.

**References:**

1. Kaushal JB, Bhatia R, Kanchan RK, Raut P, Mallapragada S, Ly QP et al. Repurposing Niclosamide for Targeting Pancreatic Cancer by Inhibiting Hh/Gli Non-Canonical Axis of Gsk3β. Cancers. 2021. https://doi.org/10.3390/CANCERS13133105.

2. Siddiqui JA, Seshacharyulu P, Muniyan S, Pothuraju R, Khan P, Vengoji R, et al. GDF15 promotes prostate cancer bone metastasis and colonization through osteoblastic CCL2 and RANKL activation. Bone Res. 2022;10:1–15.

3. Kaushal JB, Popli P, Sankhwar P, Shukla V, Dwivedi A. Sonic hedgehog protects endometrial hyperplasial cells against oxidative stress via suppressing mitochondrial fission protein dynamin-like GTPase (Drp1). Free Radic Biol Med. 2018;129:582–99.

4. Parte S, Kaur AB, Nimmakayala RK, Ogunleye AO, Chirravuri R, Vengoji R, et al. Cancer-associated Fibroblast Induces Acinar-to-du ctal Cell Transdifferentiation and Pancreatic Cancer Initiation via LAMA5/ITGA4 axis. Gastroenterology. 2023. https://doi.org/10.105 3/J.GASTRO.2023.12.018.

5. Karmakar S, Rauth S, Nallasamy P, Perumal N, Nimmakayala RK, Leon F, et al. RNA poolymerase II-associated factor 1 regulates stem cell features of pancreatic cancer cells, independently of the PAF1 complex, via interactions with PHF5A and DDX3. Gastroenterology. 2020;159:1898–1915.e6.

6. Deng YZ, Cai Z, Shi S, Jiang H, Shang YR, Ma N, et al. Cilia loss sensitizes cells to transformation by activating the mevalonate pathway. J Exp Med. 2018;215:177.

7. Yoon HG, Wong J. The corepressors silencing mediator of retinoid and thyroid hormone receptor and nuclear receptor corepressor are involved in agonist- and antagonist-regulated transcription by androgen receptor. Mol Endocrinol. 2006;20:1048–60.

8. Rauth S, Ganguly K, Atri P, Parte S, Nimmakayala RK, Varadharaj V, et al. Elevated PAF1-RAD52 axis confers chemoresistance to human cancers. Cell Rep. 2023. <https://doi.org/10.1016/J.CELREP.2023>.

9. Pandey P, Seshacharyulu P, Das S, Rachagani S, Ponnusamy MP, Yan Y, et al. Impaired expression of protein phosphatase 2A subunits enhances metastatic potential of human prostate cancer cells through activation of AKT pathway. Br J Cancer. 2013;108:2590–2600.

10. Mimeault M, Rachagani S, Muniyan S, Seshacharyulu P, Johansson SL, Datta K, et al. Inhibition of hedgehog signaling improves the anti-carcinogenic effects of docetaxel in prostate cancer. Oncotarget. 2015;6:3887.

11. Seshacharyulu P, Ponnusamy MP, Rachagani S, Lakshmanan I, Haridas D, Yan Y, et al. Targeting EGF-receptor(s) - STAT1 axis attenuates tumor growth and metastasis through downregulation of MUC4 mucin in human pancreatic cancer. Oncotarget. 2015;6:5164.

12. Gu X, Ma Y, Liu Y, Wan Q. Measurement of mitochondrial respiration in adherent cells by Seahorse XF96 Cell Mito Stress Test. STA R Protoc. 2021;2:100245.
